# Graph latent diffusion-based molecular representation learning for enhanced generalization in molecular property prediction

**DOI:** 10.1186/s13321-026-01176-8

**Published:** 2026-03-16

**Authors:** Daiki Koge, Naoaki Ono, Takashi Abe, Shigehiko Kanaya

**Affiliations:** 1 Department of Electrical and Information Engineering, Graduate School of Science and Technology, Niigata University, Ikarashi, Niigata, 950-2181 Japan; 2 Faculty of Information and Communication Engineering, Osaka Electro-Communication University, Neyagawa-shi, Osaka 572-8530 Japan; 3 Division of Information Science, Graduate School of Science and Technology, Nara Institute of Science and Technology, Ikoma, Nara 630-0192 Japan

**Keywords:** Molecular representation, Generalization performance, Transformer, Denoising diffusion probabilistic model, Latent diffusion model

## Abstract

This study aims to evaluate the effect of latent diffusion models on molecular representation learning from the perspective of generalization performance in molecular property prediction. To this end, we formulate a deep generative model for molecular representation learning based on a latent diffusion–based prior distribution, and introduce an evaluation methodology of generalization for learned molecular representations using the widely applicable information criterion (WAIC) and the widely applicable Bayesian information criterion (WBIC). Furthermore, we propose an analysis framework based on smoothness and multi-modality to analyze the factor of generalization in molecular representations. We constructed the graph latent diffusion autoencoder (Graph LDA), a deep molecular generative model that combines a transformer-based graph variational autoencoder and latent-diffusion-based latent prior distribution, designed to construct graph-level molecular representations through unsupervised learning. We compared the generalization performance of Graph LDA with other molecular representation learning models using WBIC and WAIC across multiple molecular properties, including HOMO energy, solubility, and biological activities. The results demonstrate that molecular representations learned by different models exhibit distinct generalization behaviors, and that representations learned by Graph LDA—using a latent diffusion–based prior—consistently show improved generalization in molecular property prediction. Using our proposed framework, we empirically demonstrate that the superior generalization performance of Graph LDA is attributable to the smoothness and multimodality of its learned molecular latent representation. These findings provide a principled understanding of the role of latent diffusion–based molecular representation learning in improving generalization performance.

**Scientific contribution**: This work systematically analyzed the effect of latent diffusion–based priors in molecular representation learning from the perspective of generalization performance in molecular property prediction. Through generalization evaluation using WBIC and WAIC, together with an analysis framework for molecular representations, it is empirically demonstrated that latent diffusion–based priors contribute to deep generative models extracting smooth and multimodal latent representations, which in turn lead to enhanced generalization performance of molecular representations. These findings offer a principled guideline for developing molecular representation learning models with high generalization.

**Supplementary Information:**

The online version contains supplementary material available at 10.1186/s13321-026-01176-8.

## Introduction

A molecular descriptor is a numerical representation of molecular structure, commonly used in cheminformatics to predict molecular properties such as solubility, toxicity, and bioactivity. Traditional descriptors, such as molecular fingerprints [1, 2], which represent chemical substructures as bit-strings, often result in high-dimensional and sparse representations. In contrast, graph neural networks (GNNs) leverage deep learning to automatically design molecular representations by encoding a molecular graph (graph data format of molecular structures) into a multi-dimensional feature vector [3–5]. In this manner, GNNs can construct a low-dimensional and meaningful molecular representation, enhancing the generalization performance of molecular property prediction. GNNs typically build an encoder model via supervised learning, requiring a large number of labeled molecules. However, such labeled datasets are often limited owing to the high cost of synthesizing and testing compounds, leading to overfitting and limiting the generalization performance of molecular property prediction.

Deep generative models, particularly those based on autoencoders [6] like Variational Autoencoders (VAEs) [7] offer a promising approach to address these data scarcity challenges through unsupervised learning. These autoencoders consist of two networks: an encoder that maps a molecular structure into a low-dimensional latent vector (also termed latent representation), and a decoder that generates the original molecular structure from the latent vector. During the training phase, these models can extract meaningful and continuous molecular latent representation by minimizing a combined objective, typically comprising the reconstruction error and KL-divergence. The reconstruction error measures the discrepancy between the original input molecular structure and the structure generated by the decoder, indicating how well the latent representation captures essential information required to reproduce the input. Meanwhile, KL-divergence regularizes the encoder by aligning its latent vector space with a prior distribution (e.g., standard normal), which encourages the model to learn more continuous latent representations. In practice, molecular structures are typically expressed either as Simplified Molecular Input Line Entry System (SMILES) strings [8], which represent molecules as character sequences, or as molecular graphs [9–11].

While most deep generative models for molecules are built on VAEs, a fundamental limitation of the VAEs lies in their use of simple prior distributions such as the standard normal distribution. Choosing an overly simplistic prior like the standard normal distribution for the latent representation could lead to over-regularization and posterior collapse, resulting in poor latent representations and low-quality generated samples [12, 13]. To address this problem, recent research efforts have focused on enhancing the quality of generated samples from VAEs by using learnable and flexible prior distributions [14–17]. In particular, an emerging approach involves leveraging diffusion models [18, 19]—which have shown remarkable success in high-quality image generation [20, 21]—to model the prior distribution for the latent vectors of VAEs. In this context, we refer to these models as latent diffusion VAEs. For instance, latent score-based generative model (LSGM) [16] and diffusion prior VAE [17] apply diffusion models to the latent space of convolutional neural network (CNN)-based VAEs, achieving high performance in image generation tasks.

In recent years, an increasing number of diffusion models specifically designed for molecular data have been proposed. These studies primarily focus on molecular generation using diffusion models, aiming at generating chemically valid molecular graphs [22, 23] or molecules with desired properties in extrapolative regions of the training data, such as out-of-distribution (OOD) settings [24]. In addition, several studies have reported improved performance of molecular generation by applying diffusion models to the latent space of VAEs [25, 26]. In particular, Bian et al. proposed the Hierarchical Graph Latent Diffusion Model (HGLDM) [26], an advanced approach that applies diffusion models to the latent space of a graph VAE and jointly trains the diffusion model and the encoder. Beyond evaluating the quality of generated molecules, their work also partially assesses the quality of learned molecular representations through latent space interpolation. However, in these existing studies, the evaluation of latent representations is largely limited to auxiliary analyses supporting evaluation of molecular generation tasks. To the best of our knowledge, there is no prior work that systematically investigates the effect of diffusion models on molecular representation learning for molecular property prediction, particularly from the perspective of generalization performance and the impact on the qualities of the latent representation (e.g., its smoothness and multimodality).

In this study, we aim to systematically analyze the effect of latent diffusion models on molecular representation learning from the perspective of generalization performance in molecular property prediction and how latent diffusion models influence the qualities of learned molecular representations. To this end, we formulate a molecular representation learning model based on the latent diffusion VAEs and rigorously evaluate its generalization performance using the widely applicable information criterion (WAIC) and the widely applicable Bayesian information criterion (WBIC). Furthermore, by constructing an analytical framework to assess the learned latent representations based on their smoothness and multimodality, we investigate how diffusion models influence the qualities of learned molecular representations, thereby elucidating the mechanisms by which latent diffusion contributes to the generalization of molecular representations. An advantage of adopting the latent diffusion VAE framework lies in the explicit use of diffusion as a prior distribution of the molecular latent representations in VAEs. By comparing generalization performance with deep generative models that employ simpler priors or non priors, such as the standard normal distribution, the role of diffusion in molecular representation learning can be systematically analyzed within a unified VAE-based framework. Additionally, we introduce a multi-stage training pipeline to enhance the convergence of molecular representation learning with latent diffusion VAE.

To clarify how this work differs from existing studies on diffusion models for molecular data and latent diffusion VAEs, Table 1 summarizes and compares the primary goals, diffusion model formulations, and evaluation targets of the respective studies. In the “Diffusion Model” column, when a latent diffusion model is employed, the backbone VAE architecture on which the diffusion prior is defined is explicitly indicated in parentheses. In contrast to existing diffusion-based molecular generative models, which primarily focus on conditional and unconditional molecular generation or extrapolation ability in out-of-distribution settings, this work focuses on learning molecular representations via a latent diffusion VAE and analyzes their generalization, as well as how the latent diffusion contributes to generalization. Moreover, unlike latent diffusion VAEs originally developed in the image domain to improve sample quality, our approach leverages diffusion-based prior within a permutation-invariant graph VAE (PIG-VAE) [11] framework designed for molecular representation learning to systematically analyze its effect on learned latent representations and their generalization. Importantly, we emphasize that this work does not aim to propose a novel diffusion-based molecular generative model, but rather to provide a new evaluation perspective and systematic empirical analysis of latent diffusion–based molecular representation learning.

**Table 1:** Comparison between existing studies on diffusion model and this work

Study	Primary goal	Diffusion model	Evaluation target
LSGM [16]	Image generation	Continuous latent diffusion as VAE’s prior (CNN-based VAE)	Quality of generated images (NLL, FID [27], etc.)
Diffusion prior VAE [17]	Image generation	Continuous latent diffusion as VAE’s prior (CNN-based VAE)	Quality of generated images (NLL, FID, etc.)
GDSS [22]	Molecular generation	Continuous diffusion in molecular graph space	Quality of generated molecules (validity, FCD [28], etc.)
MOOD [24]	Conditional OOD molecular generation	Continuous diffusion in molecular graph space	Extrapolation ability (properties of generated molecules, novel hit ratio, etc.)
DiGress [23]	Molecular generation	Discrete diffusion in molecular graph space	Quality of generated molecules (validity, KL divergence, etc.)
HGLDM [26]	Conditional molecular generation	Continuous latent diffusion in VAE latent space (hierarchical graph VAE)	Quality of generated molecules (properties of generated molecules, etc.); quality of latent space (e.g., continuity)
This work	Generalization analysis of molecular representation via latent diffusion	Continuous latent diffusion as VAE’s prior (PIG-VAE [11])	Generalization performance (WAIC, WBIC); quality of latent space (smoothness and multimodality)

For methods that apply diffusion in a VAE latent space, the backbone VAE architecture is specified in parentheses in the “Diffusion Model” column (e.g., CNN-based VAE or PIG-VAE). In HGLDM, diffusion is jointly trained with the encoder in the latent space and is not used as an explicit prior distribution

Notably, this study was focused on learning molecular representations from two-dimensional (2D) molecular graphs, which are easily available in most public chemical databases. While 3D molecular structures can provide useful information for molecular property prediction, they are often unavailable in many real-world datasets. Furthermore, generating accurate 3D conformations typically incurs high computational cost when using methods such as molecular dynamics simulations or quantum chemical calculations. From the perspective of scalability, extracting generalized latent representations directly from 2D molecular graphs is therefore essential for broadly applicable molecular property prediction.

We constructed a graph latent diffusion autoencoder (Graph LDA), a deep molecular generative model that combines a transformer-based graph variational autoencoder with a latent diffusion–based prior distribution, to learn graph-level molecular representations. We evaluated the generalization performance of Graph LDA by comparing its molecular property predictions with those of other molecular representation learning models using WAIC and WBIC. The results indicate that Graph LDA achieves superior generalization performance compared to the baseline models. Additionally, through our analysis framework applied to the learned latent representations, we empirically demonstrated that the latent diffusion–based prior enhances the quality of molecular latent representations in terms of smoothness and multimodality, thereby improving generalization performance.

The remainder of this paper is organized as follows. "Methods" section introduces the research methodologies, including the description of Graph LDA, the evaluation methods for generalization performance, and the analysis framework for learned molecular latent representations. "Experimental setup" section describes the experimental setup, including the datasets, baseline models used for comparison, and implementation details. "Results" section reports the generalization performance of Graph LDA and baseline models, followed by an empirical analysis of the learned latent representations. "Discussion" section discusses the implications of our findings and potential limitations. "Conclusion" section provides concluding remarks and highlights future research directions.

## Methods

This section first presents the model description of Graph LDA, including the key notation, an overview of the model, its architecture, the formulation of the latent diffusion prior for molecular representation learning, and the objective function with the training procedure ("Model description" Section). "Evaluation method of generalization performance using WAIC and WBIC" section describes the evaluation methodology for assessing generalization performance using WAIC and WBIC. "Quantitative analysis framework for molecular latent representations" section introduces an analysis framework for learned molecular latent representations.

### Model description

#### Molecular graph

A molecular graph is a data format that represents compounds as an undirected graph. A molecular graph $\mathcal {G}=(\mathcal {V}, \mathcal {E})$ is defined by node features $\mathcal {V}=\left\{ \boldsymbol{v}_1, \ldots , \boldsymbol{v}_n\right\}$ and edge features $\mathcal {E}=\left\{ \boldsymbol{e}_{i j}|(i, j) \subseteq | \mathcal {V}|\times |\mathcal {V}|\}\right.$ that connects them. Feature vectors typically include discrete entities, such as types of atoms/bonds, number of bonded hydrogens, and presence or absence of aromaticity. GNNs are commonly used for extracting features of molecular graphs as low-dimensional latent representations.

#### Overview of Graph LDA

Graph LDA is a deep molecular generative model that combines a transformer-based graph VAE and denoising diffusion probabilistic model (DDPM) [18].

**Fig. 1:**
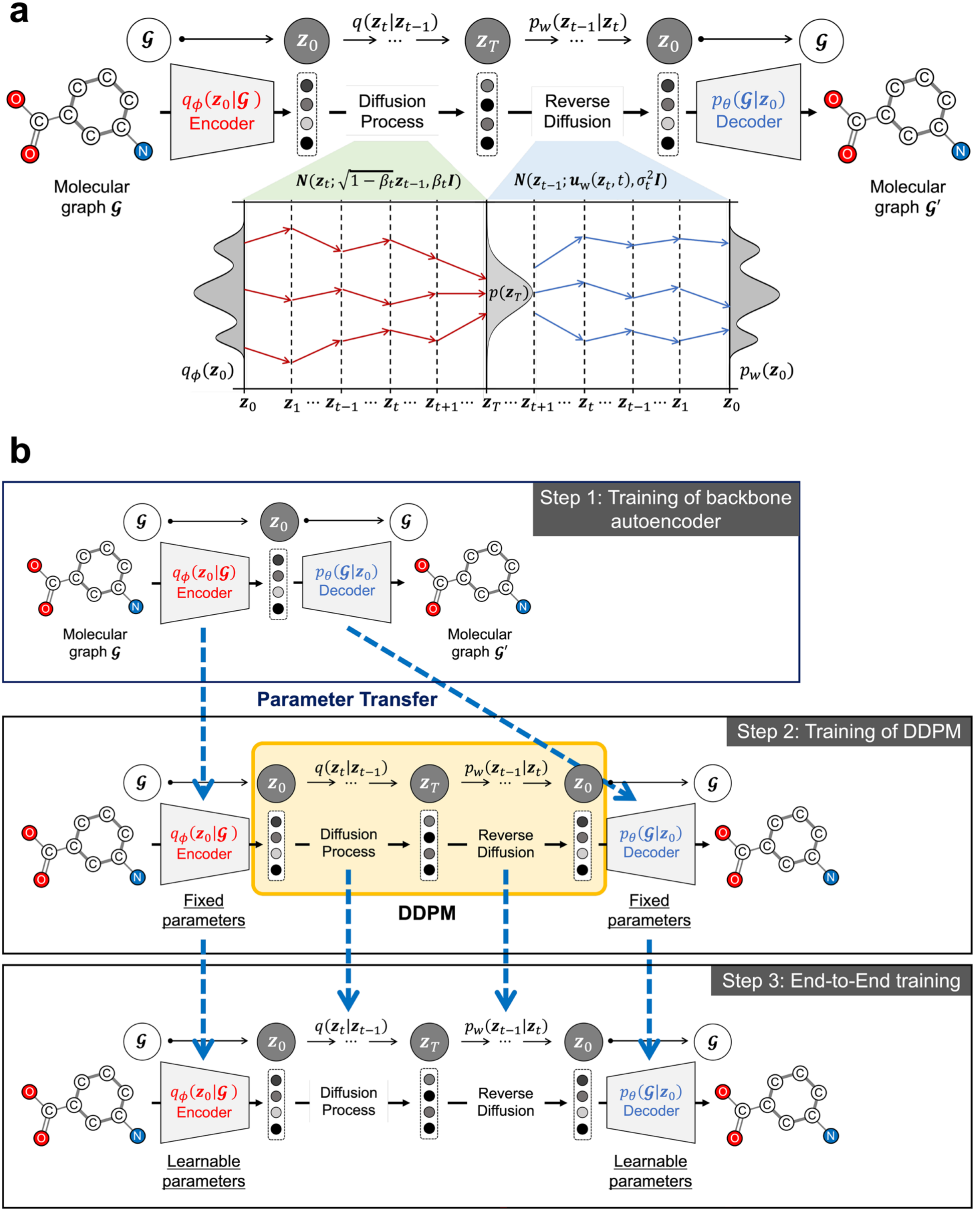
Overview of Graph LDA. **a** The whole Graph LDA framework: Graph LDA consists of graph VAE’s encoder, diffusion process, reverse diffusion process, and graph VAE’s decoder. **b** Multi-stage training strategy: Graph LDA is trained in three stages—(1) pre-training of the backbone graph VAE for molecular reconstruction, (2) training of the DDPM on the latent vector space, and (3) end-to-end fine-tuning integrating all components for efficient optimization

An overview of the Graph LDA is shown in Fig. 1. Graph LDA consists of an encoder, a diffusion process, a reverse diffusion process, and a decoder. The encoder of graph VAE maps a molecular graph $\mathcal {G}$ into a latent vector $\boldsymbol{z}_0$ following a multivariate normal distribution $q_\phi \left( \boldsymbol{z}_0 \mid \mathcal {G}\right)$. The diffusion process of DDPM progressively transforms the latent vector $\boldsymbol{z}_0$ into a noise vector $\boldsymbol{z}_T \sim \mathcal {N}(\textbf{0}, \boldsymbol{I}) \equiv p\left( \boldsymbol{z}_T\right)$, which follows a standard normal distribution, by adding a noise $\boldsymbol{\varepsilon } \sim N(\textbf{0}, \boldsymbol{I})$. The reverse diffusion process of DDPM progressively recovers the noise vector to the original latent vector $\boldsymbol{z}_0$ from the encoder model. The decoder of graph VAE maps the latent vector $\boldsymbol{z}_0$ into the original molecular graph $\mathcal {G}$.

We further introduce a multi-stage training strategy, as illustrated in Fig. 1b. In this strategy, Graph LDA is trained in three stages to ensure sufficient convergence and to effectively impose regularization induced by the latent diffusion prior. First, the backbone autoencoder (the encoder and decoder of graph VAE) is pre-trained to establish a stable molecular graph reconstruction capability. Next, the DDPM is trained on the latent vectors obtained from the encoder to model a smooth and structured latent distribution. Finally, all components are fine-tuned jointly in an end-to-end manner to integrate the encoder, diffusion process, and decoder. This progressive training scheme allows the model to effectively learn both molecular reconstruction and latent space regularization within a unified framework. A formal description of the multi-stage training strategy is provided in "Training strategy" Section.

#### Model architecture

This section describes all components of the Graph LDA.

**Permutation-invariant graph variational autoencoder (PIG-VAE)**

The biological and physico-chemical properties of a molecule depend on not only its local molecular structure but also its global structural information. Learning meaningful representations of complete molecular graphs is a fundamental problem for molecular property prediction for drug and material discovery [3, 4, 29].

In this study, we use the PIG-VAE [11], which maps a molecular graph into a single latent vector that represents both the global and local information. PIG-VAE is an encoder–decoder model for learning latent representations of molecular graphs that are invariant to the permutations of atoms. The details of the encoder and decoder architectures are described in the Supplementary material.

**Latent diffusion**

Latent diffusion is a framework that applies DDPM to the latent space of VAEs. DDPM is a hierarchical deep generative model that learns the reverse operation of diffusion, a stochastic process that progressively destroys information. The diffusion process for latent vectors $\boldsymbol{z}_0$ sampled from the encoder $q_\phi \left( \boldsymbol{z}_0 \mid \mathcal {G}\right)$ is defined as follows.

1 $$\begin{aligned} \begin{aligned} q\left( \boldsymbol{z}_{1: T} \mid \boldsymbol{z}_0\right) =\prod _{t=1}^T q\left( \boldsymbol{z}_t \mid \boldsymbol{z}_{t-1}\right) , \\ q\left( \boldsymbol{z}_t \mid \boldsymbol{z}_{t-1}\right) =\mathcal {N}\left( \boldsymbol{z}_t ; \sqrt{1-\beta _t} \boldsymbol{z}_{t-1}, \beta _t \boldsymbol{I}\right) . \end{aligned} \end{aligned}$$

We assume that the variance schedule $\beta _0, \ldots , \beta _T$ linearly increases from $\beta _1 = 0.001$ to $\beta _T = 0.2$. Defining $\alpha _t=1-\beta _t$, $\bar{\alpha }_t:=\prod _{s=1}^t \alpha _s$, and $\bar{\beta }_t=1-\bar{\alpha }_t$, $\boldsymbol{z}_t$ at an arbitrary time step *t* can be sampled as

2 $$\begin{aligned} q\left( \boldsymbol{z}_t \mid \boldsymbol{z}_0\right) =\mathcal {N}\left( \boldsymbol{z}_t ; \bar{\alpha }_t \boldsymbol{z}_0,\bar{\beta }_t \boldsymbol{I}\right) . \end{aligned}$$

In contrast to the diffusion process, reverse diffusion aims to recover the original latent vector $\boldsymbol{z}_0$ from the degraded latent representation $\boldsymbol{z}_T$. The learnable denoising kernel is denoted as

3 $$\begin{aligned} \begin{aligned} p_w\left( \boldsymbol{z}_{0: T}\right)&=p\left( \boldsymbol{z}_T\right) \prod _{t=1}^T p_w\left( \boldsymbol{z}_{t-1} \mid \boldsymbol{z}_t\right) , \\ p_w\left( \boldsymbol{z}_{t-1} \mid \boldsymbol{z}_t\right)&=\mathcal {N}\left( \boldsymbol{z}_{t-1} ; \mu _w\left( \boldsymbol{z}_t, t\right) , \sigma _t^2 \boldsymbol{I}\right) . \end{aligned} \end{aligned}$$

The joint distribution $p_w\left( \boldsymbol{z}_{0: T}\right)$ corresponds to the reverse diffusion process, and it is defined as a Markov chain with learnable Gaussian transitions starting at $p\left( \boldsymbol{z}_T\right) =\mathcal {N}\left( \boldsymbol{z}_T; 0, \boldsymbol{I}\right)$.

#### Latent diffusion VAE with DDPM as prior

Graph LDA is a latent diffusion VAE for molecular representation learning using latent diffusion-based prior distribution.

**Fig. 2:**
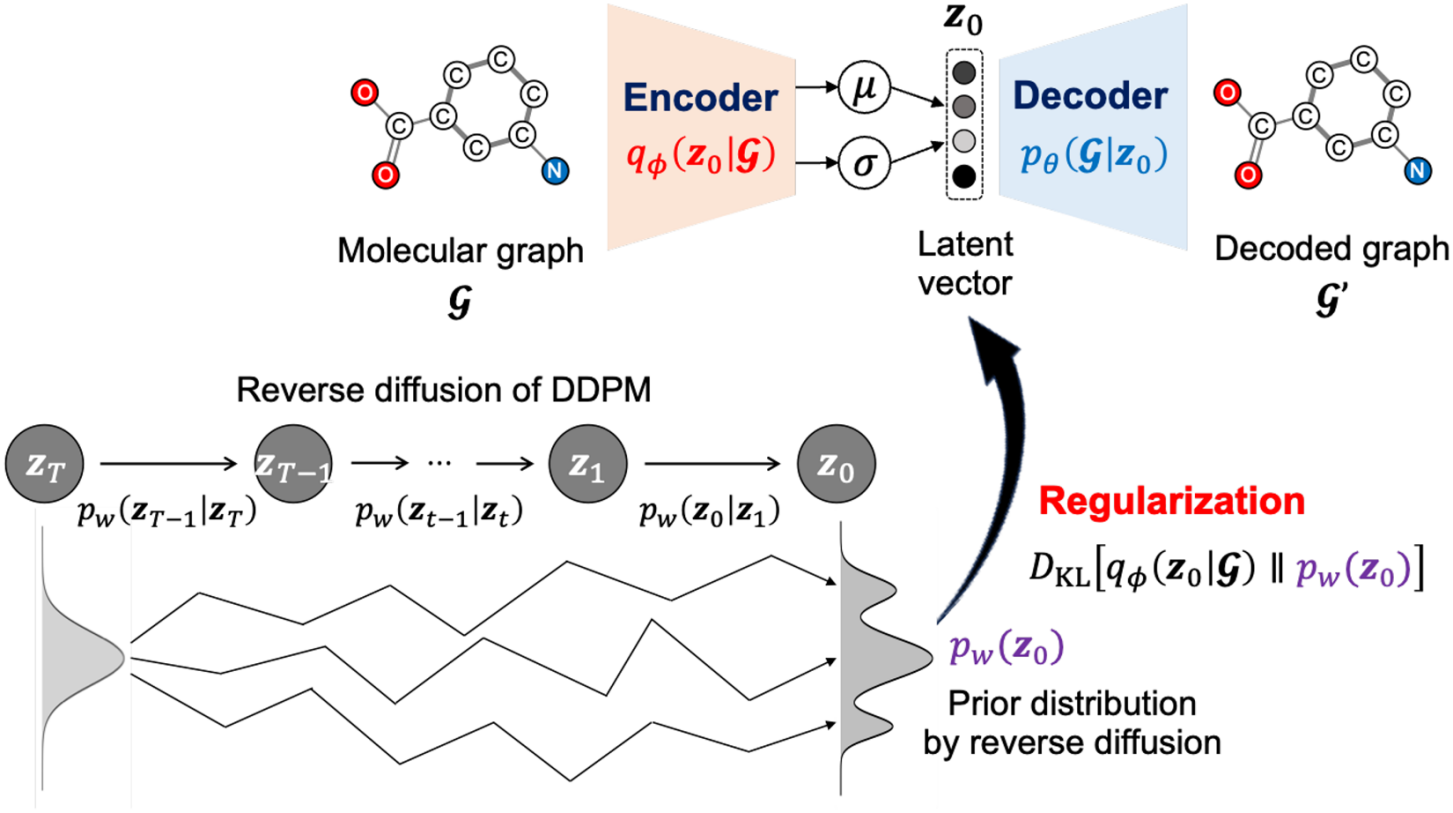
Graphical model of Graph LDA

The objective of Graph LDA is to maximize the log marginal likelihood for the observed data (molecular graph $\mathcal {G}$). We define the log marginal likelihood and evidence lower bound (ELBO) using the marginal distribution $p_w\left( \textbf{z}_0\right)$ by the reverse diffusion of DDPM as a prior distribution on the latent vector $\boldsymbol{z}_0$.

4 $$\begin{aligned} \log p_\theta (\mathcal {G})&= \log \int p_\theta \left( \mathcal {G} \mid \boldsymbol{z}_0\right) p_w\left( \boldsymbol{z}_0\right) d \boldsymbol{z}_0 \end{aligned}$$

5 $$\begin{aligned}&\ge \mathbb {E}_{q_\phi \left( \boldsymbol{z}_0 \mid \mathcal {G}\right) }\left[ \log p_\theta (\mathcal {G} \mid \boldsymbol{z}_0)\right] -D_{\textrm{KL}}\left[ q_\phi \left( \boldsymbol{z}_0 \mid \mathcal {G}\right) \Vert p_w\left( \boldsymbol{z}_0\right) \right] \end{aligned}$$

From the above two equations (4) and (5), the latent prior model $p_w\left( \boldsymbol{z}_0\right)$ contributes to maximizing the marginal likelihood $p_\theta (\mathcal {G})$ as a learnable and flexible prior distribution, and also contributes to regularizing the encoder $q_\phi (\boldsymbol{z}_0 \mid \mathcal {G})$ (Fig. 2). This enables the model to learn more expressive and continuous molecular latent representations.

We consider $\log p_w\left( \textbf{z}_0\right)$ as the log marginal likelihood of $\boldsymbol{z}_0$ and derive the lower bound of Eq. (5) as follows:

6 $$\begin{aligned} \begin{aligned} \mathbb {E}_{q_\phi \left( \boldsymbol{z}_0 \mid \mathcal {G}\right) } \Biggl [ \log p_\theta (\mathcal {G} \mid \boldsymbol{z}_0) - \log q_\phi \left( \boldsymbol{z}_0 \mid \mathcal {G}\right) + \mathbb {E}_{q\left( \boldsymbol{z}_{1:T} \mid \boldsymbol{z}_0\right) } \biggl [\log \frac{p_w\left( \boldsymbol{z}_{0:T}\right) }{q(\boldsymbol{z}_{1:T} \mid \boldsymbol{z}_0)}\biggr ] \Biggr ]. \end{aligned} \end{aligned}$$

The following expression is obtained, given $D_{\textrm{KL}}\left[ q\left( \boldsymbol{z}_T \mid \boldsymbol{z}_0\right) \Vert p\left( \boldsymbol{z}_T\right) \right] \simeq 0$:

7 $$\begin{aligned} \begin{aligned} \mathbb {E}_{q_\phi \left( \boldsymbol{z}_0 \mid \mathcal {G}\right) } \Biggl [ \underbrace{\log p_\theta (\mathcal {G} \mid \boldsymbol{z}_0)}_{\text {i. reconstruction}} + \underbrace{\bigl ( - \log q_\phi \left( \boldsymbol{z}_0 \mid \mathcal {G}\right) \bigr )}_{\text {ii. entropy}} + \underbrace{\mathbb {E}_{q\left( \boldsymbol{z}_1 \mid \boldsymbol{z}_0\right) } \biggl [\log p_w(\boldsymbol{z}_0 | \boldsymbol{z}_1)\biggr ]}_{\text {iii. latent reconstruction}} \\ - \underbrace{\sum _{t>1} \mathbb {E}_{q\left( \boldsymbol{z}_t \mid \boldsymbol{z}_0\right) }\biggl [D_{\textrm{KL}}\left[ q\left( \boldsymbol{z}_{t-1} \mid \boldsymbol{z}_t, \boldsymbol{z}_0\right) \Vert p_w\left( \boldsymbol{z}_{t-1} \mid \boldsymbol{z}_t\right) \right] \biggr ]}_{\text {iv. latent denoising}} \Biggr ]. \end{aligned} \end{aligned}$$

Terms (i) and (ii) denote the log-likelihood of the data $\mathcal {G}$, determined using the PIG-VAE decoder $p_w\left( \mathcal {G} \mid \boldsymbol{z}_0\right)$ and entropy of the PIG-VAE encoder $q_\phi \left( \boldsymbol{z}_0 \mid \mathcal {G}\right)$, respectively. The other terms are defined as follows.

**iii. Latent reconstruction:** This term represents the log-likelihood of predicting a latent vector $\boldsymbol{z}_0 \sim q_\phi \left( \boldsymbol{z}_0 \mid \mathcal {G}\right)$ from $\boldsymbol{z}_1 \sim q\left( \boldsymbol{z}_1 \mid \boldsymbol{z}_0\right)$. We define the prediction model $p_w(\boldsymbol{z}_0 | \boldsymbol{z}_1)$ as the following conditional normal distribution:

8 $$\begin{aligned} \begin{aligned} p_w(\boldsymbol{z}_0 | \boldsymbol{z}_1) = \mathcal {N}\left( \boldsymbol{z}_{0} ; \mu _w\left( \boldsymbol{z}_1\right) , \sigma _1^2 \boldsymbol{I}\right) , \\ \mu _w\left( \boldsymbol{z}_1\right) = \frac{1}{\sqrt{\alpha _1}}\boldsymbol{z}_1, \ \sigma _1^2 = \beta _1. \end{aligned} \end{aligned}$$

We obtain $\boldsymbol{z}_1 \sim q\left( \boldsymbol{z}_1 \mid \boldsymbol{z}_0\right)$ by reparametrizing Eq. (2) as $\boldsymbol{z}_1 = \sqrt{\alpha _1}\boldsymbol{z}_0 + \sqrt{\beta _1}\boldsymbol{\epsilon }$ for $\boldsymbol{\epsilon } \sim \mathcal {N}(\textbf{0}, \boldsymbol{I})$. Thus, the latent reconstruction term can be rewritten as follows:

9 $$\begin{aligned} \mathbb {E}_{q\left( \boldsymbol{z}_1 \mid \boldsymbol{z}_0\right) } \biggl [\log p_w(\boldsymbol{z}_0 | \boldsymbol{z}_1)\biggr ] = \mathbb {E}_{\boldsymbol{\epsilon } \sim \mathcal {N}(\textbf{0}, \boldsymbol{I})}\Biggl [C_1 - \frac{\boldsymbol{\epsilon }^2}{2\alpha _1}\Biggr ], \end{aligned}$$

where $C_1 = \log \Bigl (1/\sqrt{2\pi }\beta _1\Bigr )$. Thus, term (iii) is a constant during training and can be ignored.

**iv. Latent denoising:** Following [18], after simplifications, term (iv) can be rewritten as follows:

10 $$\begin{aligned} \mathbb {E}_{t \sim \textbf{U}(1,T)}\left[ \left\| \boldsymbol{\epsilon }-\epsilon _w\left( \boldsymbol{z}_t, t\right) \right\| ^2\right] , \end{aligned}$$

where $\boldsymbol{\epsilon } \sim \mathcal {N}(\textbf{0}, \boldsymbol{I})$. $\epsilon _w$, named the denoising model, is a trainable multilayer perceptron with skip connections. The latent vector at time step *t* is sampled by $\boldsymbol{z}_t=\sqrt{\bar{\alpha }_t} \boldsymbol{z}_0+\sqrt{\bar{\beta }_t} \boldsymbol{\epsilon }$ for $\boldsymbol{\epsilon } \sim \mathcal {N}(\textbf{0}, \boldsymbol{I})$. Details of the denoising model architecture are provided in the Supplementary Material.

#### Objective function

The objective function for model training is established as follows:

11 $$\begin{aligned} \begin{aligned} \mathbb {E}_{q_\phi \left( \boldsymbol{z}_0 \mid \mathcal {G}\right) } \Biggl [\log p_\theta (\mathcal {G} \mid \boldsymbol{z}_0) + \bigl ( - \log q_\phi \left( \boldsymbol{z}_0 \mid \mathcal {G}\right) \bigr ) - \mathbb {E}_{t \sim \textbf{U}(1,T)}\left[ \left\| \boldsymbol{\epsilon }-\epsilon _w\left( \boldsymbol{z}_t, t\right) \right\| ^2\right] \Biggr ], \end{aligned} \end{aligned}$$

where $$\boldsymbol{\epsilon } \sim \mathcal {N}(\textbf{0}, \boldsymbol{I})$$ and $$\boldsymbol{z}_t=\sqrt{\bar{\alpha }_t} \boldsymbol{z}_0+\sqrt{\bar{\beta }_t} \boldsymbol{\epsilon }$$.

#### Training strategy

To enhance the stability and convergence performance of Graph LDA training, we introduce a multi-stage training algorithm. A formal description of the training process is provided in Algorithm 1. In the following sections, the Graph LDA model trained with this multi-stage strategy is denoted as Graph LDA (stable).

**Algorithm 1:**
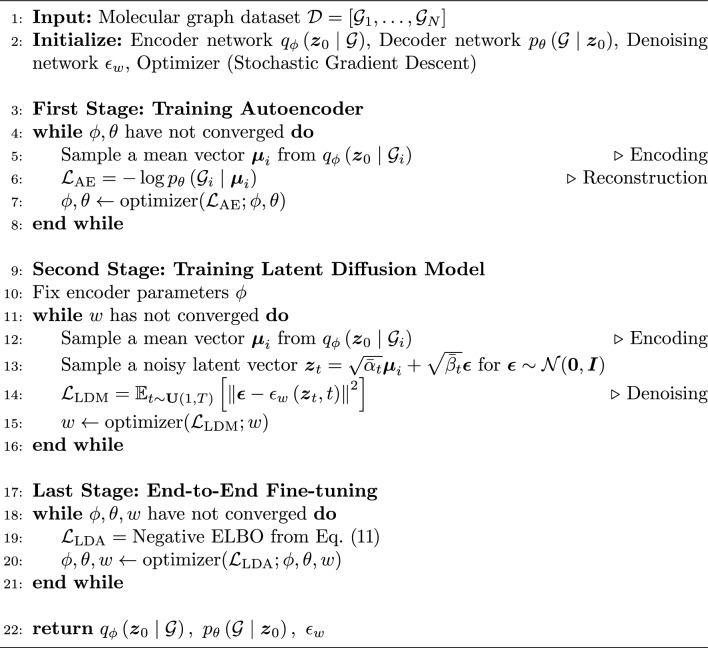
Multi-Stage training scheme for Graph LDA

### Evaluation method of generalization performance using WAIC and WBIC

We analyze the generalization performance of the learned molecular representations in predicting molecular properties using WAIC [30] and WBIC [31]. Unlike commonly used evaluation methods based on hold-out and cross-validation for prediction models, which are sensitive to data partitioning and often suffer from high bias for estimating generalization performance, WAIC and WBIC provide theoretically grounded, unbiased estimators of generalization performance. Furthermore, these information criteria are particularly suitable for singular statistical models such as deep neural networks, where the assumptions underlying traditional model selection criteria such as Akaike’s information criterion (AIC) [32] and Bayesian information criteria (BIC) [33] do not hold.

In this section, we first describe the formulation of the prediction models using a pre-trained molecular encoder, and then explain the computation of WAIC and WBIC.

#### Bayesian neural network

**Fig. 3:**
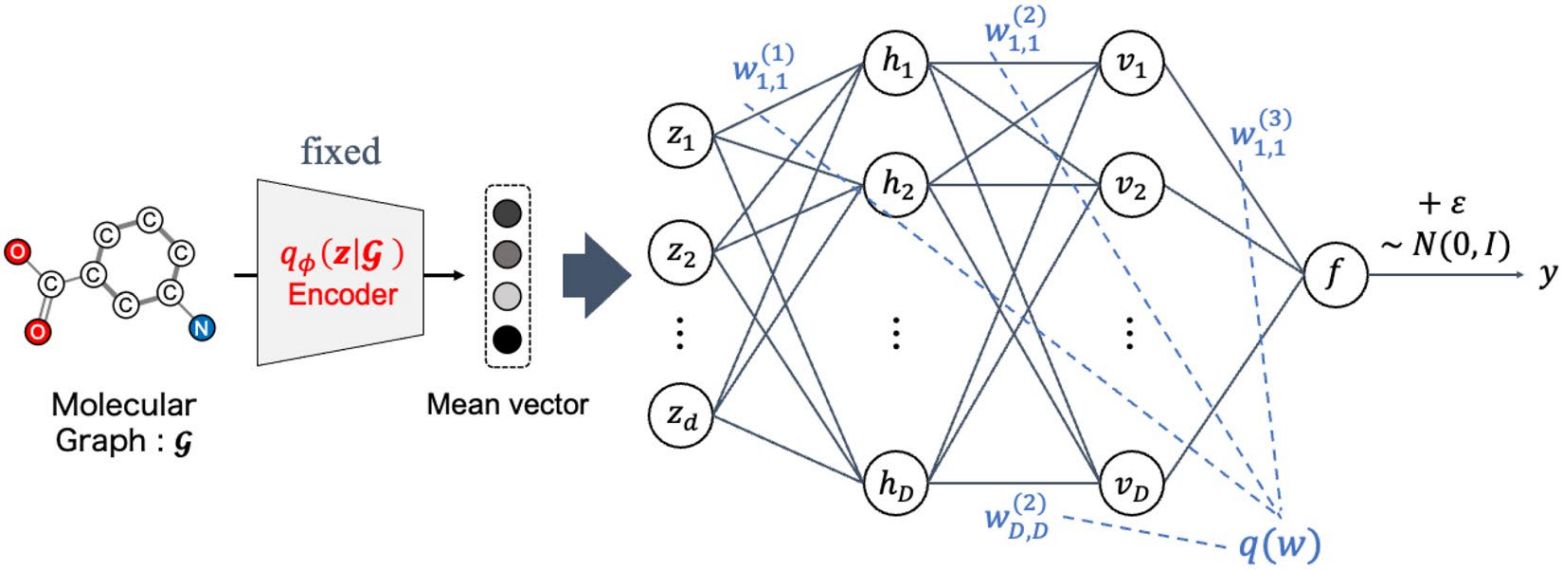
Scheme of Bayesian neural network

A stochastic regression model based on Bayesian neural networks is constructed, and its information criteria are computed. The model is represented by a probability density function $p(y \mid \boldsymbol{w}, \hat{\phi }, \mathcal {G})$, where $\hat{\phi }$ are pre-trained parameters of the encoder $: \mathcal {G} \rightarrow \boldsymbol{z} \in \mathbb {R}^{\textrm{d}}$ and remain fixed. $\boldsymbol{w}$ are regression parameters, and the prior probability density function is $q(\boldsymbol{w})$. The pre-trained encoder represents the conditional normal distribution $q_\phi (\boldsymbol{z} \mid \mathcal {G})$ and models its mean $\boldsymbol{\mu }$ and variance $\boldsymbol{\sigma }$ vectors. The conditional distribution $p(y \mid \boldsymbol{w}, \hat{\phi }, \mathcal {G})$ is used to derive the molecular property value *y* from the encoded mean vector $\boldsymbol{\mu }$ obtained by the pre-trained encoder from the molecular graph $\mathcal {G}$. The Bayesian neural network predicts molecular properties using the mean vector $\boldsymbol{\mu }$ as the input (Fig. 3).

#### WAIC

Assume that the observed samples $(\boldsymbol{y}^N, \boldsymbol{\mathcal {G}}^N) = [(y_1, \mathcal {G}_1), (y_2, \mathcal {G}_2), \ldots , (y_N, \mathcal {G}_N)]$ are independently subjected to the probability density function $q^*(y, \mathcal {G}) = q^*(y \mid \mathcal {G})q^*(\mathcal {G})$, termed the true distribution. The joint probability density function is

12 $$\begin{aligned} q^*(\boldsymbol{y}^N, \boldsymbol{\mathcal {G}}^N)=\prod _{i=1}^N q^*(y_i, \mathcal {G}_i). \end{aligned}$$

The generalization error, one of the measures of generalization performance, is the expectation value of the negative log-likelihood of the prediction model for the true distribution and is defined by the following equation:

13 $$\begin{aligned} G=-\int \int q^*(y, \mathcal {G}) \log p\left( y \mid \mathcal {G}, \hat{\phi }, \boldsymbol{y}^N, \boldsymbol{\mathcal {G}}^N \right) dy d\mathcal {G}, \end{aligned}$$

14 $$\begin{aligned} p\left( y \mid \mathcal {G}, \hat{\phi }, \boldsymbol{y}^N, \boldsymbol{\mathcal {G}}^N \right) =\int p\left( y \mid \boldsymbol{w}, \hat{\phi }, \mathcal {G}\right) p\left( \boldsymbol{w} \mid \hat{\phi }, \boldsymbol{y}^N, \mathcal {G}^N\right) d\boldsymbol{w}. \end{aligned}$$

Although the generalization error *G* cannot be computed when the true distribution $q^*(y, \mathcal {G})$ is unknown, WAIC provides an unbiased estimation of the expectation $\mathbb {E}[G]$ with a joint distribution of the true distribution. $\mathbb {E}[G]$ and WAIC are defined as follows:

15 $$\begin{aligned} \mathbb {E}[G]&= -\int \int q^*(\boldsymbol{y}^N, \boldsymbol{\mathcal {G}}^N) \Bigl [ \int \int q^*(y, \mathcal {G}) \log p\left( y \mid \mathcal {G}, \hat{\phi }, \boldsymbol{y}^N, \boldsymbol{\mathcal {G}}^N \right) dy\, d\mathcal {G} \Bigr ] d\boldsymbol{y}^N\, d\boldsymbol{\mathcal {G}}^N. \end{aligned}$$

16 $$\begin{aligned} \text {WAIC}&= - \frac{1}{N} \sum _{i=1}^{N}\log p\left( y_i \mid \mathcal {G}_i, \hat{\phi }, \boldsymbol{y}^N, \boldsymbol{\mathcal {G}}^N \right) \nonumber \\&\quad + \frac{1}{N} \sum _{i=1}^N\left\{ \mathbb {E}_{\boldsymbol{w}}\left[ \left( \log p(y_i \mid \boldsymbol{w}, \hat{\phi }, \mathcal {G}_i)\right) ^2\right] -\mathbb {E}_{\boldsymbol{w}}\left[ \log p(y_i \mid \boldsymbol{w}, \hat{\phi }, \mathcal {G}_i)\right] ^2\right\} , \end{aligned}$$

where $\mathbb {E}_{\boldsymbol{w}}[\cdot ]$ is the expectation value with Bayesian posterior $p(\boldsymbol{w} \mid \hat{\phi }, \boldsymbol{y}^N, \boldsymbol{\mathcal {G}}^N) \propto \prod _{i=1}^N p(y_i \mid \boldsymbol{w}, \mathcal {G}_i, \hat{\phi }) q(\boldsymbol{w})$, defined as

17 $$\begin{aligned} p\left( y_i \mid \mathcal {G}_i, \hat{\phi }, \boldsymbol{y}^N, \boldsymbol{\mathcal {G}}^N \right) =\int p\left( y_i \mid \boldsymbol{w}, \hat{\phi }, \mathcal {G}_i\right) p\left( \boldsymbol{w} \mid \hat{\phi }, \boldsymbol{y}^N, \mathcal {G}^N\right) d\boldsymbol{w}. \end{aligned}$$

#### WBIC

Bayesian free energy $\mathcal {F}$ is another measure of generalization performance, defined as

18 $$\begin{aligned} - \log p(\boldsymbol{y}^N \mid \hat{\phi }, \boldsymbol{\mathcal {G}}^N) = -\log \int \prod _{i=1}^N p\left( y_i \mid \boldsymbol{w}, \hat{\phi }, \mathcal {G}_i\right) q(\boldsymbol{w}) d\boldsymbol{w}. \end{aligned}$$

The corresponding expectation value is

19 $$\begin{aligned} \mathbb {E}[\mathcal {F}] = -\int \int q^*(\boldsymbol{y}^N, \boldsymbol{\mathcal {G}}^N) \Bigl [\log p(\boldsymbol{y}^N \mid \hat{\phi }, \boldsymbol{\mathcal {G}}^N) \Bigr ] d\boldsymbol{y}^N d\boldsymbol{\mathcal {G}}^N. \end{aligned}$$

The Bayesian free energy is an unbiased estimator for the expectation value $\mathbb {E}[\mathcal {F}]$ with the joint distribution of the true distribution $q^*(y, \mathcal {G})$. Minimizing $\mathbb {E}[\mathcal {F}]$ is equivalent to minimizing the Kullback–Leibler divergence from $q^*\left( \boldsymbol{y}^N, \boldsymbol{\mathcal {G}}^N\right)$ to $p(\boldsymbol{y}^N, \boldsymbol{\mathcal {G}}^N \mid \hat{\phi }) = p(\boldsymbol{y}^N \mid \hat{\phi }, \boldsymbol{\mathcal {G}}^N)q^*\left( \boldsymbol{\mathcal {G}^N}\right)$. WBIC is an approximation of the Bayesian free energy $\mathcal {F}$ and defined as

20 $$\begin{aligned} \text {WBIC} = \mathbb {E}_{\boldsymbol{w}}^\beta \left[ \sum _{i=1}^{N}-\log p\left( y_i \mid \boldsymbol{w}, \hat{\phi }, \mathcal {G}_i\right) \right] , \quad \beta = 1/\log {N}, \end{aligned}$$

where $\mathbb {E}_{\boldsymbol{w}}^\beta [\cdot ]$ is the expectation value with Bayesian posterior $p_\beta (\boldsymbol{w} \mid \hat{\phi }, \boldsymbol{y}^N, \boldsymbol{\mathcal {G}}^N) \propto \prod _{i=1}^N p(y_i \mid \boldsymbol{w}, \mathcal {G}_i, \hat{\phi })^\beta q(\boldsymbol{w})$.

#### Posterior sampler

WAIC and WBIC can be calculated using the posterior sampler, with Langevin Monte Carlo (LMC) [34] applied in this study. In its standard form, LMC updates iterations as

21 $$\begin{aligned} \boldsymbol{w}_{k+1}=\boldsymbol{w}_k- \frac{\eta ^2}{2} \nabla U\left( \boldsymbol{w}_k\right) + \eta \boldsymbol{\xi }_k, \end{aligned}$$

where $\boldsymbol{\xi }_k \sim \mathcal {N}\left( \textbf{0}, I\right)$ is a Gaussian random vector, and $U\left( \boldsymbol{w}\right)$ is the potential function, defined as follows:

22 $$\begin{aligned} U(\boldsymbol{w})=-\log q(\boldsymbol{w})- \sum _{i=1}^N\log p\left( y_i \mid \boldsymbol{w}, \hat{\phi }, \mathcal {G}_i\right) . \end{aligned}$$

The hyperparameters for the Bayesian neural networks and Langevin Monte Carlo (LMC) used in our experiments are provided in the Supplementary Material.

### Quantitative analysis framework for molecular latent representations

This section introduces a quantitative analysis framework for evaluating the multimodality and smoothness of the learned molecular latent representations.

#### Multimodality in molecular latent representation

We quantify the multimodality in the latent distribution based on the number of clusters determined using the X-means algorithm [35]. X-means models each cluster as a Gaussian distribution and optimizes the BIC to determine the number of clusters. A higher number of clusters indicates a greater degree of multimodality in the latent representation.

X-means first performs clustering using k-means [36]. However, clustering algorithms based on the distance between samples, such as k-means, typically exhibit poor performances in high-dimensional datasets [37, 38]. To address this problem, we introduce a practical scheme integrating the dimensionality reduction method and X-means to estimate the number of clusters in the molecular latent representation. The scheme involves the following two steps.

**Step 1.** The dimension of the molecular latent representation is reduced using the uniform manifold approximation and projection (UMAP) method [39]. UMAP is a nonlinear dimensionality reduction method that maps high-dimensional data to a low-dimensional space while preserving the k-nearest neighbor points in the high-dimensional data space. To ensure that this mapping retains crucial information regarding the molecular latent representation, we determine the dimension (*l*) of the low-dimensional space by analyzing the kNN preserving rate (kNN-rate).

23 $$\begin{aligned} \text {kNN-rate}=\frac{1}{N \times k} \sum _{i=1}^N\left| \mathcal {N}^\text {high}_k(i) \cap \mathcal {N}^\text {low}_k(i)\right| , \end{aligned}$$

where $\mathcal {N}^\text {high}_k(i)$ and $\mathcal {N}^\text {low}_k(i)$ denote the *k*-nearest neighbors of the *i*-th molecule in the high-dimensional and low-dimensional spaces, respectively. We set k to 0.5% of the total sample size of the entire dataset. We compute the kNN-rate for each unsupervised learning model as *l* ranges from 1 to *d* (dimension of the molecular latent representation) and determine the dimension of the low-dimensional space for UMAP using the kneedle algorithm [40].

**Step 2.** Following the dimensionality reduction of the latent representation using UMAP with $\hat{l}$ determined in **Step 1**, the number of clusters is obtained using X-means. Although the X-means framework reported by Pelleg and Moore [35] assumes an isotropic Gaussian distribution for each cluster, we apply a multivariate normal distribution using the unbiased covariance of samples within a cluster. Additionally, clusters with sample sizes below *k* are excluded.

We use the number of clusters *k* determined by this scheme as a proxy measure to assess the multimodality of the molecular latent representation.

#### Local smoothness in molecular latent representations

The smoothness is quantified by the gradient norm of the molecular property values. We compute the average of the directional derivative norm of the molecular property for several directions at the latent vector point $\boldsymbol{a}$ of an anchor molecule $\mathcal {M(\boldsymbol{a})}$. The average of the directional derivative norm along a vector $\textbf{v}$ at an anchor latent point $\boldsymbol{a}$, determined using the finite difference method with a scalar value $\epsilon$, is

24 $$\begin{aligned} \mathbb {E}_{\textbf{v}}\left[ \left\| \nabla _{\textbf{v}} f(\boldsymbol{a})\right\| \right] \simeq \frac{1}{M}\sum _{i=1}^M \left\| \frac{f(\boldsymbol{a}+\epsilon \textbf{v}_i)-f(\boldsymbol{a})}{\epsilon }\right\| , \end{aligned}$$

25 $$\begin{aligned} \nabla _{\textbf{v}} f(\boldsymbol{a})=\lim _{h \rightarrow 0} \frac{f(\boldsymbol{a}+h \textbf{v})-f(\boldsymbol{a})}{h}, \end{aligned}$$

where $f: \boldsymbol{a} \in \mathbb {R}^d \rightarrow \mathbb {R}$ is an unknown molecular property function. We select a molecule from the molecular dataset corresponding to the neighborhood point $\boldsymbol{u}_i$ ($\equiv \boldsymbol{a}+\epsilon \textbf{v}_i$) of the anchor latent point $\boldsymbol{a}$, based on the Euclidean distance in the latent space, and set its molecular property value $f(\boldsymbol{u}_i)$ as $f(\boldsymbol{a}+\epsilon \textbf{v}_i)$. Then, Eq. (24) can be rewritten with $\mathcal {N}(\boldsymbol{a})$ as a set of neighborhood points of $\boldsymbol{a}$ as follows:

26 $$\begin{aligned} \mathbb {E}_{\textbf{v}}\left[ \left\| \nabla _{\textbf{v}} f(\boldsymbol{a})\right\| \right] \simeq \frac{1}{M}\sum _{\boldsymbol{u}_i \in \mathcal {N}(\boldsymbol{a})}^M \left\| \frac{f(\boldsymbol{u}_i)-f(\boldsymbol{a})}{\epsilon }\right\| \end{aligned}$$

$(f(\boldsymbol{u}_i)-f(\boldsymbol{a})) / \epsilon$ is a directional derivative of $f(\boldsymbol{a})$ along a vector $(\boldsymbol{u}_i - \boldsymbol{a}) / \epsilon$. In this study, we set $\epsilon = 1.0$, with 5 or 10 neighborhood points. We evaluate the average value of this mean directional derivative norm for several anchor molecules. A lower average value indicates that the molecular latent representation is smooth for a particular molecular property.

To further assess local smoothness, we analyze the Tanimoto similarity of extended-connectivity fingerprints (ECFP) [2]. For anchor molecules, we select 50 neighboring points in the latent vector space based on distance and calculate their Tanimoto similarity to the anchor molecule. We finally evaluate the local smoothness by averaging the Tanimoto similarity values for all anchor molecules. This metric also reflects the local smoothness of the molecular latent representation, with higher average values indicating smoother representations.

## Experimental setup

This section describes the dataset used for training, comparison models, and training process.

### Construction of molecular latent representations

We use the ZINC-250k dataset from the ZINC-15 database [41] and QM9 dataset [42] for constructing molecular latent representations. ZINC-250k is a subset of the ZINC-15 database that contains 250,000 organic compounds. These datasets are widely adopted in molecular representation learning, as they consist of chemically valid compounds with a diverse yet balanced distribution. We selected these datasets because the stability and effectiveness of deep learning model training are highly dependent on the quality of the molecular data, including structural diversity and the absence of extreme outliers or distributional bias.

**Fig. 4:**
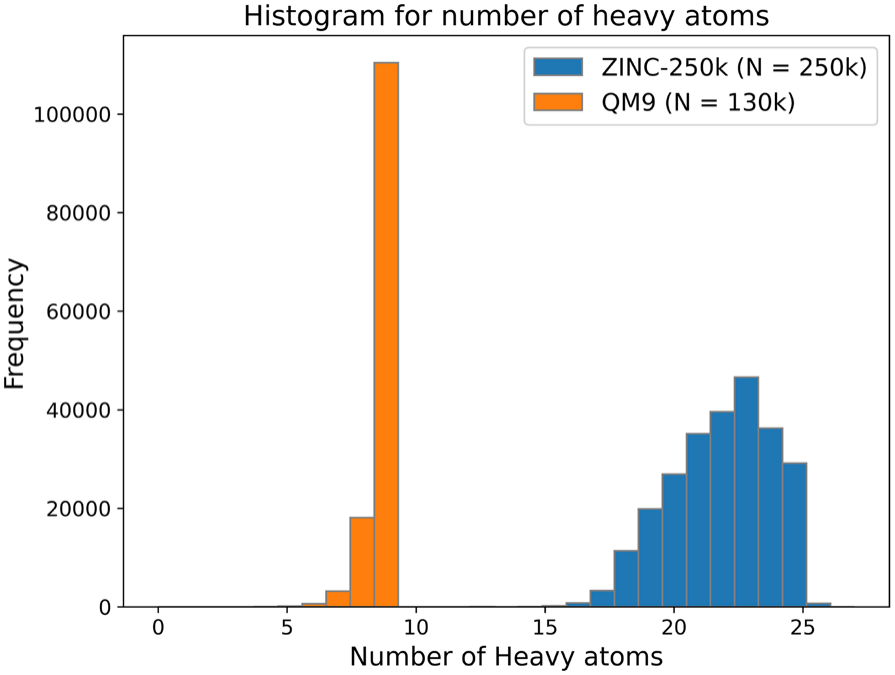
Molecular weight histogram of ZINC-250k and QM9

Both datasets include organic compounds with low molecular weight, with different distributions for the number of heavy atoms. (Fig. 4).

#### Definition of molecular graphs

ZINC-250k and QM9 also differ in terms of the type of atoms in the molecule samples. Thus, we define different molecular graphs for each dataset and construct molecular latent representations for each dataset. Specifically, molecular graphs are defined by feature vectors for each atom and bond. The definitions of molecular graphs for ZINC-250k and QM9 are presented in Tables 2 and 3, respectively.

**Table 2:** Atom and bond features of ZINC-250k

Indices	Description	Options	Type
Atom features			
0–11	Atom identity	C, N, O, F, Br, Si, P, S, Cl, I, B, other	One-hot
12–22	Degree	0, 1, 2, 3, 4, 5, 6, 7, 8, 9, 10	One-hot
23–29	Implicit valence	0, 1, 2, 3, 4, 5, 6	One-hot
30	Formal charge	$\mathbb {R}$	Value
31	Number of radical electrons	$\mathbb {R} \ge 0$	Value
32–36	Hybridization	*sp*, $sp^2$, $sp^3$, $sp^3d$, $sp^3d^2$	One-hot
37	Aromaticity	*true*, *false*	0 or 1
38–42	Implicit hydrogen	0, 1, 2, 3, 4	One-hot
Bond features			
0–4	Bond type	Single, double, triple, aromatic, none	One-hot
5	Conjugation	*True*, *false*	0 or 1
6	In ring	*true*, *false*	0 or 1
7–12	Topological distance	$0 \le \mathbb {R} \le 5$	One-hot

**Table 3:** Atom and bond features of QM9

Indices	Description	Options	Type
Atom features			
0–4	Atom identity	C, N, O, F, H	One-hot
5–15	Degree	0, 1, 2, 3, 4, 5, 6, 7, 8, 9, 10	One-hot
16–22	Implicit valence	0, 1, 2, 3, 4, 5, 6	One-hot
23	Formal charge	$\mathbb {R}$	Value
24	Number of radical electrons	$\mathbb {R} \ge 0$	Value
25–29	Hybridization	*sp*, $sp^2$, $sp^3$, $sp^3d$, $sp^3d^2$	One-hot
30	Aromaticity	*True*, *false*	0 or 1
31–35	Implicit hydrogen	0, 1, 2, 3, 4	One-hot
Bond features			
0–4	Bond type	Single, double, triple, aromatic, no bond	One-hot
5	Conjugation	*True*, *false*	0 or 1
6	In ring	*True*, *false*	0 or 1
7	Euclidean distance	$\mathbb {R} \ge 0$	Value

#### Baseline models

In the experiments, we compare the generalization performance of the Graph LDA with several baseline models for molecular representation learning. These include classical unsupervised deep generative models such as a graph autoencoder (Graph AE), graph variational autoencoder (Graph VAE), and graph normalizing flow (Graph Flow). Graph VAE is a graph generative model based on VAEs [7], and Graph Flow adopts a normalizing-flow-based autoencoder structure [43]. VAE and normalizing flow are representative unsupervised deep learning models for learning molecular latent representation [9, 10, 44, 45].

Additionally, to perform a more comprehensive evaluation, we include recent self-supervised learning (SSL) methods such as MolCLR [46], GraphMVP [47], and 3D-Infomax [48] as strong baselines. These models leverage contrastive learning and auxiliary tasks to learn a graph encoder model that extracts molecular representations from 2D molecular graphs. For GraphMVP and 3D-Infomax, we use the pre-trained models provided by their official repositories. This is because these methods incorporate molecular conformation data during representation learning, which are not included in the ZINC-250k and QM9 datasets.

All unsupervised learning models (Graph AE, VAE, Flow, and Graph LDA) use the same autoencoder backbone (PIG-VAE). The SSL models are implemented using official repositories with hyperparameters recommended in the original papers.

#### Hyperparameters

Table 4 summarizes the hyperparameters of the autoencoder backbone (PIG-VAE) for unsupervised learning models.

**Table 4:** Hyperparameters

Description	Final choice
Dimension of latent vectors	90 (for ZINC-250k), 50 (for QM9)
Number of encoder layers	16
Dimension of message vectors (encoder)	256
Attention head (encoder)	16
Dimension of positional encoding vectors	64
Number of decoder layers	16
Dimension of message vectors (decoder)	256
Attention head (decoder)	16
Batch size	20
Optimizer	Adam [49]
Learning rate (lr)	0.00005
Scheduler	ExponentialLR ($\gamma = 0.9$)

Each unsupervised learning model is trained on a dataset with $N=50$k samples for 100 epochs, and the model with the best performance is trained on the validation dataset ($N=10$k samples). For Graph LDA implemented with the multi-stage training scheme, the first training stage involves 100 epochs, second stage involves 500 epochs, and last stage involves 100 epochs with lr $= 0.00001$.

### Datasets for constructing molecular property prediction model

We establish regression models using a constructed molecular representation for various types of continuous molecular properties and evaluate their generalization performance. This design choice was made to evaluate the model’s generalization under realistic noise conditions. Specifically, regression tasks typically contain more label noise than classification tasks, rendering them a more stringent and meaningful testbed for generalized representation learning.

We use the following molecular property datasets for the assessment.

- BACE: IC50 (half maximal inhibitory concentration) values of compounds for $\beta$-secretase from ExCAPE-DB [50].
- CTSD: IC50 values of compounds for cathepsin D from ExCAPE-DB.
- MMP2: IC50 values of compounds for Matrix Metallo proteinase-2 from ExCAPE-DB.
- Malaria: Log EC50 (in vitro half-maximal effective concentration) measurements of compounds against a sulfide-resistant strain of ${P. falciparum}$, the parasite that causes malaria [51].
- ESOL: Aqueous solubility values [52] of compounds.
- Lipo: Experimental results of octanol/water distribution coefficient (logD at pH 7.4) for small molecules [53].
- LogP: logP values of ZINC-250k compounds [41].
- Freesolv: Experimental and calculated hydration free energies for small molecules in water [54].
- HOMO: Highest occupied molecular orbital energy values of small compounds from QM9 dataset.
- LUMO: Lowest occupied molecular orbital energy values of small compounds from QM9 dataset.

Many of the datasets in this list, including ESOL, Freesolv and Lipo, are part of the MoleculeNet benchmark [53]. Moreover, to ensure a comprehensive evaluation of representation quality, we compile datasets spanning different levels of molecular properties, including physico-chemical properties (e.g., logP, ESOL), biological activities (e.g., malaria inhibition), and quantum chemical properties (e.g., HOMO/LUMO energy levels). This enables us to comprehensively assess the generalization of constructed molecular representations.

The architecture used in this work, PIG-VAE, requires fixing the maximum number of atoms per molecule in both training and inference. Therefore, the molecules used in each dataset are restricted to those with sizes not exceeding those of molecules in the ZINC-250k and QM9 datasets.

Given the bias in the size of molecules and number of heavy atoms in the dataset, we use the model trained on the QM9 dataset for predicting HOMO and LUMO energy levels. For the prediction of other molecular properties, models trained on the ZINC-250k dataset are used.

### Implementation

All experiments are conducted using Python 3.10.13. RDKit (version 2024.3.5.0) is used for processing the organic compound data, and PyTorch (version 2.4.0) and Pytorch Geometric (version 2.5.3) are used as the deep learning frameworks. Details of the computational environment are provided in the Supplementary Material.

## Results

This section first evaluates the generalization performance of the Graph LDA and baseline models predicting molecular properties ("Generalization performance" section). "Analysis results of learned molecular representations" section provides an empirical analysis of the molecular representations for each model, focusing on their multimodality and local smoothness by our proposed framework. Finally, "Computational efficiency and effectiveness of multi-stage training strategy" section discusses the computational efficiency and convergence behavior of the model, highlighting the effectiveness of the multi-stage training strategy.

### Generalization performance

Tables 5 and 6 summarize WAIC and WBIC values for the latent diffusion VAEs (Graph LDA and Graph LDA (stable)) and baseline models, respectively. Graph LDA (stable) is trained by multi-stage training scheme (Algorithm 1), whereas Graph LDA is trained by joint-training scheme. Overall, Tables 5 and 6 show that Graph LDA (stable) consistently achieves low WAIC and WBIC values across many molecular property prediction tasks, indicating best or near-best generalization performance. Furthermore, when restricting the comparison to autoencoder-based unsupervised learning models (i.e., Graph AE, Graph VAE, and Graph Flow), Graph LDA (stable) consistently achieves lower WAIC and WBIC values than these models across most tasks.

In addition, we analyze the significant differences in generalization performance among the models using the Bayes factor. The Bayes factor enables a comparison of two competing statistical models based on their marginal likelihoods (evidences). We evaluate the Bayes factor from the marginal likelihoods for the observed data obtained from two different encoder models ($\hat{\phi }_0, \hat{\phi }_1$). For a given dataset $\left( \boldsymbol{y}^N, \boldsymbol{\mathcal { G }}^N\right) =\left[ \left( y_1, \mathcal {G}_1\right) ,\left( y_2, \mathcal {G}_2\right) , \ldots ,\left( y_N, \mathcal {G}_N\right) \right]$, the plausibility of the two different encoder models $\hat{\phi }_0$ and $\hat{\phi }_1$ is assessed using the Bayes factor $\text {BF}_{01}$ as

27 $$\begin{aligned} \textrm{BF}_{01}=\frac{p(\boldsymbol{y}^N \mid {\hat{\phi}}_0, \boldsymbol{\mathcal {G}^N)}}{p(\boldsymbol{y}^N \mid {\hat{\phi}}_1, \boldsymbol{\mathcal {G}^N)}}=\exp \left( \mathcal {F}_1-\mathcal {F}_0\right) , \end{aligned}$$

where $\mathcal {F}_0$ is the WBIC value of the Graph LDA (stable), and $\mathcal {F}_1$ is WBIC value of the baseline models. The results of the Bayes factor analysis are presented in Table 7. According to the interpretation guidelines of the Bayes factor provided by Kass and Raftery [55] (Table 8), Graph LDA (stable) is characterized by “strong evidence” for 5 out of 10 molecular properties.

**Table 5:** Comparison of WAIC values between Graph LDA and baseline models

Model	Pre-trained by ZINC-250k								Pre-trained by QM9	
	BACE (502)	CTSD (84)	MMP2 (1046)	Malaria (3019)	ESOL (1080)	Freesolv (642)	Lipo (1903)	LogP (5000)	HOMO (5000)	LUMO (5000)
Graph AE	1.6527	1.3127	1.9250	1.9307	1.9694	6.5067	1.5808	1.2637	0.9197	0.9202
Graph VAE	2.3065	2.7163	2.3914	2.0570	2.6324	6.3741	1.6094	1.7197	0.9194	0.9203
Graph Flow	1.9652	4.9729	2.4368	2.0415	2.7785	6.6498	1.7165	2.0326	0.9251	0.9203
Graph LDA	1.6649	1.5114	2.2960	1.9622	2.4819	6.2410	1.6742	1.5379	0.9194	**0.9201**
Graph LDA (stable)	**1.5737**	1.5975	**1.8984**	**1.8790**	1.9891	7.4011	**1.5657**	1.2606	**0.9193**	**0.9201**
MolCLR	1.8885	**1.1455**	2.5243	2.092	3.2210	15.2203	1.7054	2.2092	0.9197	0.9206
Graph MVP	1.8942	1.2833	2.2605	1.9445	**1.9157**	**5.6350**	1.6202	**1.2157**	0.9194	**0.9201**
3D-Infomax	2.0084	6.7185	2.5359	2.0745	3.0402	6.5997	1.7270	2.2050	**0.9193**	0.9202

The best generalization performance (i.e., the lowest WAIC) across all models is highlighted in bold, while the best-performing autoencoder-based unsupervised learning model is underlined

**Table 6:** Comparison of WBIC values between Graph LDA and baseline models

Model	Pre-trained by ZINC-250k								Pre-trained by QM9	
	BACE (502)	CTSD (84)	MMP2 (1046)	Malaria (3019)	ESOL (1080)	Freesolv (642)	Lipo (1903)	LogP (5000)	HOMO (5000)	LUMO (5000)
Graph AE	852.15	113.70	2095.09	**6011.00**	2238.24	2955.63	3073.68	7320.31	4607.90	4611.13
Graph VAE	1357.98	236.74	3012.55	6444.87	3126.67	3634.13	3307.00	11121.36	4607.85	4608.97
Graph Flow	1007.83	156.25	2565.11	6124.93	2896.51	4664.99	3250.75	9573.39	4602.76	4604.07
Graph LDA	851.86	122.64	2426.64	6081.41	2686.33	3829.41	3213.24	8189.20	**4601.64**	4604.51
Graph LDA (stable)	**843.67**	124.98	**2079.20**	6086.26	2223.51	**2845.08**	**3068.35**	**6532.18**	4602.16	4604.07
MolCLR	1017.82	**105.42**	2684.47	6592.34	3299.12	5326.98	3382.58	12519.20	4908.47	4778.35
Graph MVP	913.72	110.50	2350.02	6117.86	**2003.59**	3197.05	3080.24	7755.46	4610.46	4611.76
3D-Infomax	1086.72	271.43	2702.92	6265.93	3324.31	5404.93	3306.31	11033.21	4619.89	**4600.93**

The best generalization performance (i.e., the lowest WBIC) across all models is highlighted in bold, while the best-performing autoencoder-based unsupervised learning model is underlined

**Table 7:** Bayes factor (logBF) based on WBIC of Graph LDA (stable)

Model	Pre-trained by ZINC-250k								Pre-trained by QM9	
	BACE	CTSD	MMP2	Malaria	ESOL	Freesolv	Lipo	LogP	HOMO	LUMO
Graph AE	16.96	−22.56	31.78	−150.52	29.46	221.10	10.66	1576.26	11.48	14.12
Graph VAE	1028.62	223.52	1866.70	717.22	1806.32	1578.10	477.30	9178.36	11.38	9.80
Graph Flow	328.32	62.54	971.82	77.34	1346.00	3639.82	364.80	6082.42	1.20	0.00
MolCLR	348.30	−39.12	1210.54	1012.16	2151.22	4963.80	628.46	11974.04	612.62	348.56
Graph MVP	140.10	−28.96	541.64	63.20	−439.84	703.94	23.78	2446.56	16.60	15.38
3DInfomax	486.10	1247.44	292.90	359.34	2201.60	5119.70	475.92	9002.06	35.46	−6.28

**Table 8:** Interpretation of Bayes factor

$\text {2}\log _e \text {BF}_{01}$	Strength of evidence
$< 0$	Negative (supports baseline model)
0–2	Not worth more than a bare mention Graph LDA (stable)
2–6	Substantial evidence for Graph LDA (stable)
6–10	Strong evidence for Graph LDA (stable)
$> 10$	Decisive for Graph LDA (stable)

As an ablation study, we conducted experiments in which interatomic distance–based edge features were replaced with topological distances, thereby removing explicit 3D structural information. Even under this setting, Graph LDA maintained strong generalization performance and outperformed baseline models. The detailed results are provided in Supplementary Table S8. In addition to the WAIC and WBIC based generalization analysis, we also conducted an evaluation of predictive performance using a hold-out evaluation with multiple splits as an ablation analysis, as commonly employed in previous studies on molecular property prediction. We summerize the results over four independent splits in the Suppelementary Table S9.

### Analysis results of learned molecular representations

This section describes the analysis results of the multimodality and local smoothness of the learned molecular latent representations by our framework.

To isolate and understand the specific contribution of the latent diffusion-based prior to the qualities of the molecular latent representations, we focused this analysis on the deep generative models that share the same backbone architecture (PIG-VAE) but differ primarily in their regularization or prior distribution: Graph AE (without an explicit prior on the latent representations), Graph VAE and Graph Flow (using a standard normal prior), and Graph LDA (using a latent diffusion prior). While SSL models like MolCLR, GraphMVP, and 3D-Infomax are included as important performance baselines in our main generalization evaluation (Tables 5 and 6), they were intentionally excluded from this analysis.

####  Evaluation of multimodality

This section reports the analysis results of multimodality in molecular latent representations by our scheme proposed in "Multimodality in molecular latent representation" section. The kNN-rates for all unsupervised learning models and selection of $\hat{l}$ values are outlined in the Supplementary material. This scheme is implemented with 50,000 randomly selected molecules from ZINC-250k and QM9, respectively. Table 9 lists the number of clusters determined by X-means for each unsupervised learning model. For ZINC-250k, Graph AE and the latent diffusion VAEs (Graph LDA and Graph LDA (stable)) exhibit a higher number of clusters compared with Graph VAE and Graph Flow. For QM9, similar to the case of ZINC-250k, the number of clusters in Graph AE and the latent diffusion models is higher than those in Graph VAE and Graph Flow, although the difference is less pronounced.

**Table 9:** Comparison of number of clusters determined by X-means for each model

Dataset	Models				
	Graph AE	Graph VAE	Graph Flow	Graph LDA	Graph LDA (stable)
ZINC-250k	78	1	1	78	57
QM9	113	101	103	110	106

**Fig. 5:**
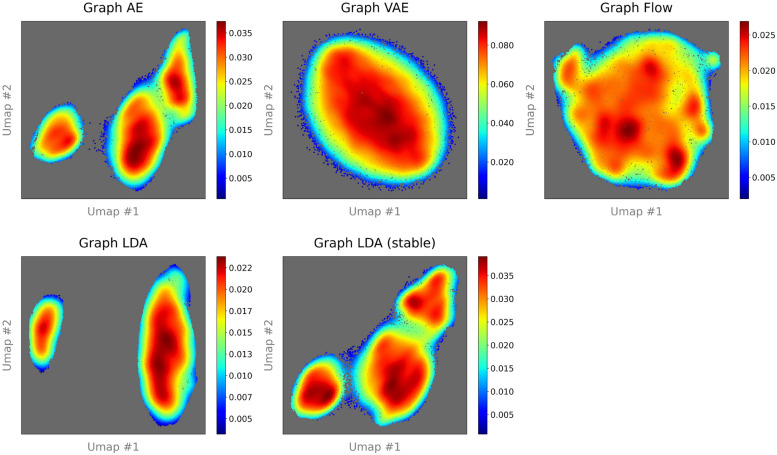
UMAP plots of chemical latent space in ZINC-250k with 50,000 molecules. The number of neighboring points is 250. Each point representing a molecule is colored with the density value based on kernel density estimation (KDE) [56]

**Fig. 6:**
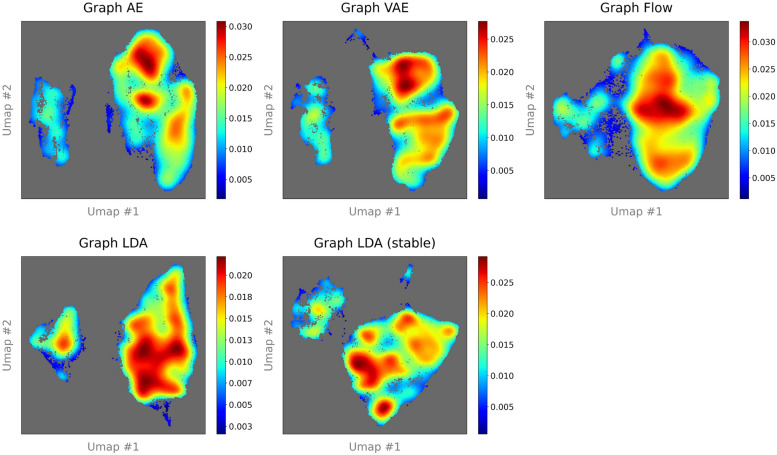
UMAP plots of chemical latent space in QM9 with 50,000 molecules. The number of neighboring points is 250. Each point representing a molecule is colored with the density value based on KDE

Figures 5 and 6 show the visualization of two-dimensional molecular latent spaces obtained by UMAP for ZINC-250k and QM9 datasets, respectively. Visualization of ZINC-250k dataset shows that Graph VAE and Graph Flow gather the entire set of molecular mappings into a single cluster. In contrast, the visualization of QM9 dataset shows that all models divide the entire set of molecular mappings into multiple clusters. These results are consistent with the results of latent cluster analysis based on X-means.

#### Evaluation of local smoothness 

This section reports the analysis results of smooothness in molecular latent representations by our scheme proposed in "Local smoothness in molecular latent representations" section. In this experiment, we set $\epsilon = 1.0$ in the calculation of the mean directional derivative norm (Eq. 26), with 10 neighborhood points for LogP, HOMO, and LUMO, and five neighborhood points for the other properties.

**Fig. 7:**
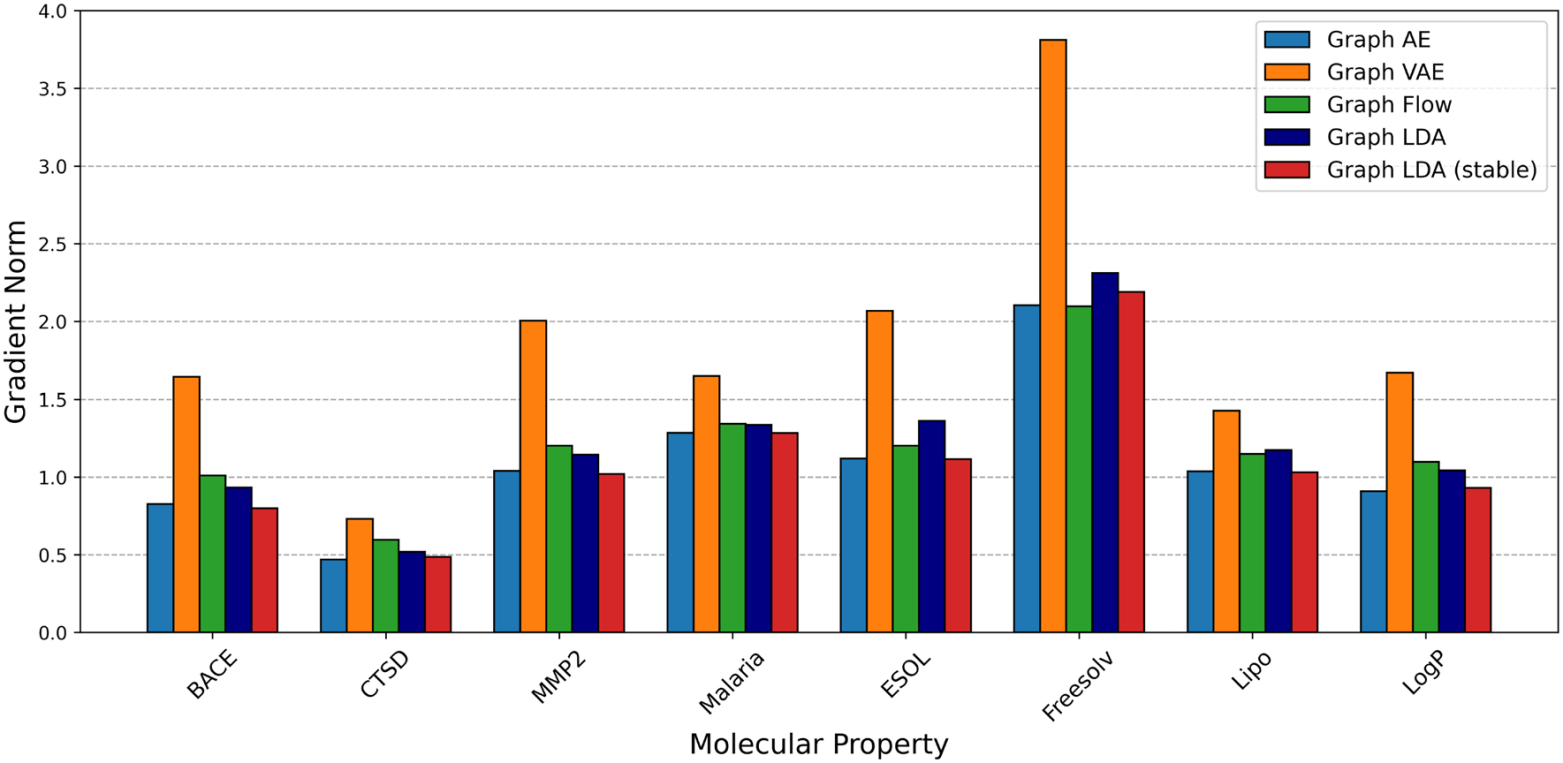
Comparison of smoothness on different models trained by ZINC-250k

**Fig. 8:**
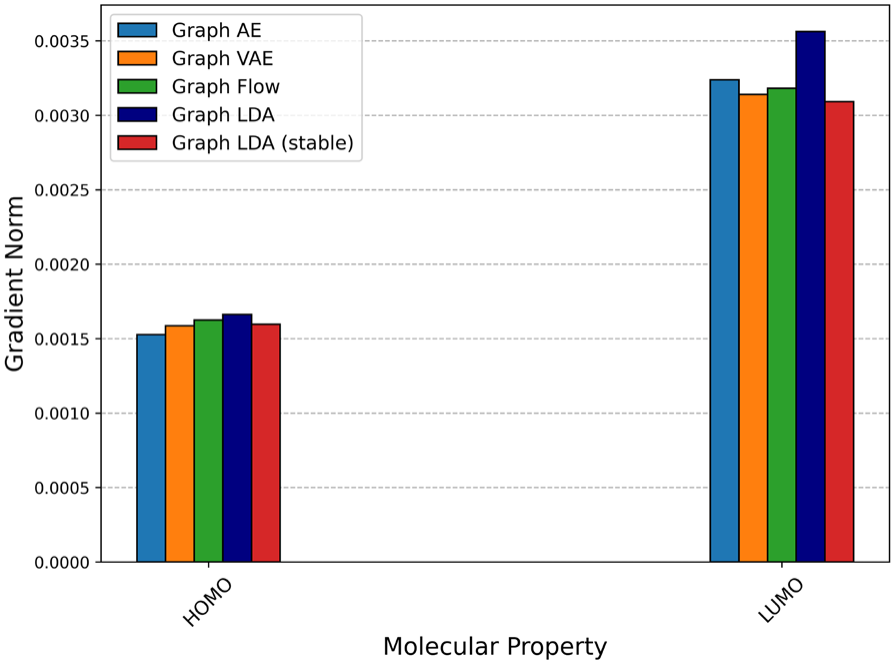
Comparison of smoothness on different models trained by QM9

Figures 7 and 8 present a comparison of the smoothness of molecular latent representations for each model trained by ZINC-250k and QM9, respectively. Graph LDA (stable) shows a lower mean directional derivative norm for 6 out of 10 molecular properties compared with the baseline models. Details of the gradient norm for each model are presented in the supplementary material.

**Fig. 9:**
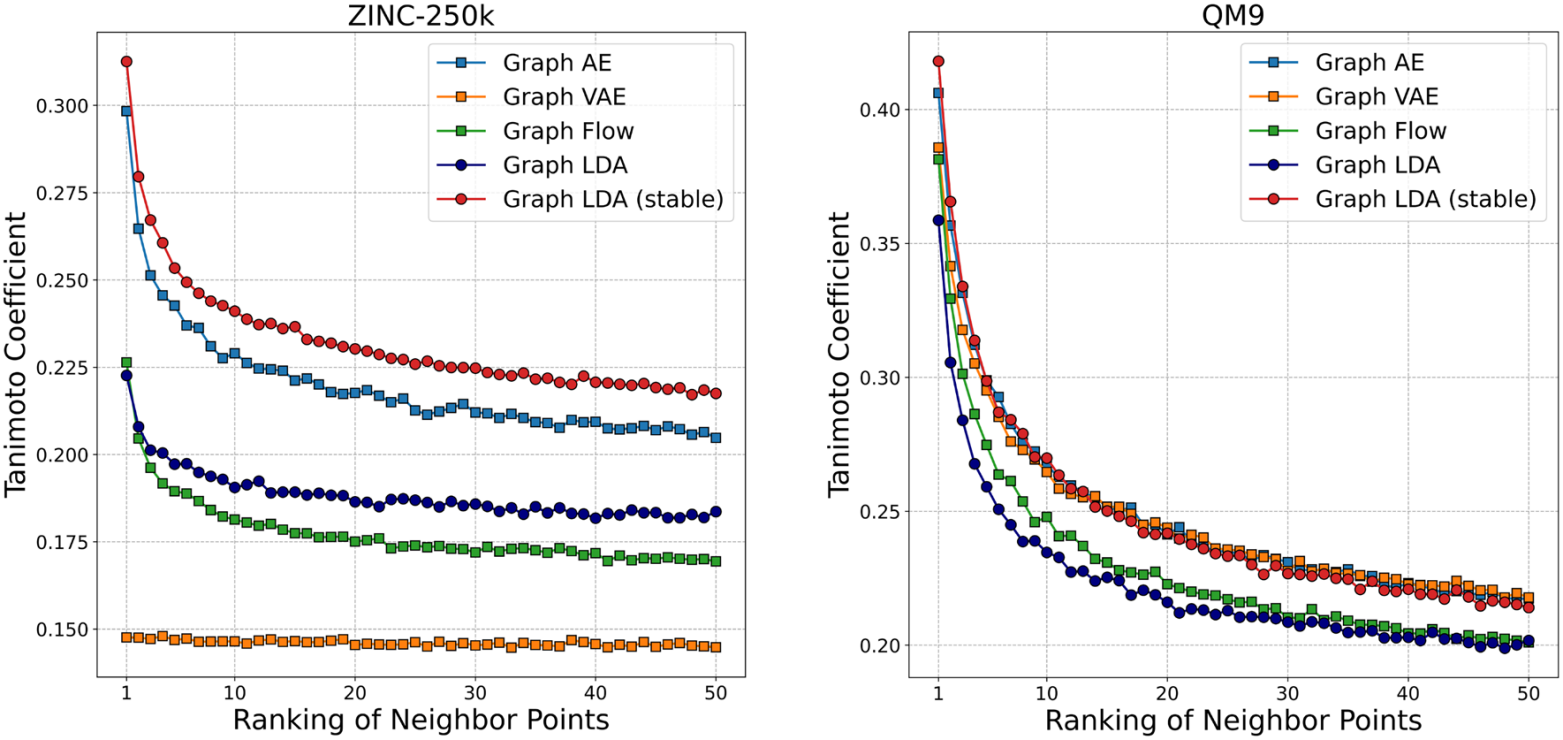
Comparison of Tanimoto coefficient using ECFP4 at neighboring points in latent vector space of each model. The horizontal axis indicates the neighbor rank (i.e., the order of proximity from the anchor point)

We also reported the smoothness analysis results by Tanimoto similarity. For 5,000 anchor molecules, we select 50 neighboring points in the latent vector space based on distance and calculate their Tanimoto similarity to the anchor molecule. Figure 9 presents the average Tanimoto similarity across all anchor molecules. For ZINC-250k, Graph LDA (stable) consistently demonstrated greater smoother than the other models across the entire range of 1 to 50 neighboring points. In contrast, for QM9, no significant differences were observed between models, except for Graph LDA and Graph Flow.

### Computational efficiency and effectiveness of multi-stage training strategy

We evaluated the computational efficiency and optimization stability of Graph LDA (stable), which employs the multi-stage training strategy. The training time per epoch and total training time are summarized in Supplementary Tables S11 and S12, for both ZINC-250k and QM9. Despite the multi-stage training process, Graph LDA achieves competitive computational efficiency, with per-epoch training time comparable to that of Graph VAE.

**Fig. 10:**
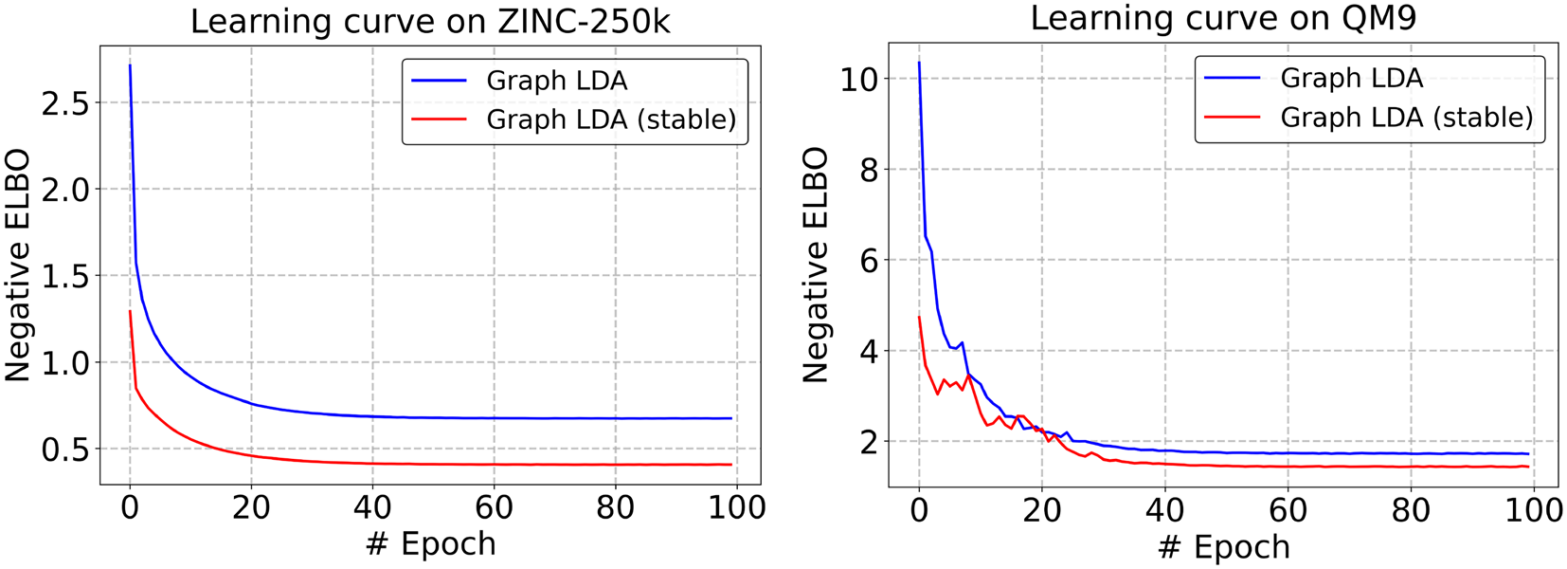
Learning curves of the joint-training model (Graph LDA) and the multi-stage training model [Graph LDA (stable)] on the ZINC-250k and QM9 datasets

To investigate the effectiveness of the multi-stage training scheme, we also compared its convergence behavior with that of the conventional joint-training approach. The convergence behavior of the multi-stage and joint-training schemes is presented in Fig. 10, which shows the learning curves of the negative ELBO during training. For the multi-stage model [Graph LDA (stable)], the learning curve corresponds to the final end-to-end fine-tuning stage described in Algorithm 1. The multi-stage model gradually converges to lower negative ELBO values than the joint-training model (Graph LDA), indicating more effective convergence. While the joint-training model exhibits smooth convergence, it tends to remain at relatively higher loss levels, suggesting limited convergence efficiency. These results demonstrate that the multi-stage training strategy improves the convergence performance and enables the model to reach lower minima without increasing computational cost (see Tables S11 and S12).

Furthermore, the comparison of generalization performance between the joint and multi-stage training models is summarized in Supplementary Table S13. The results demonstrate that the multi-stage training strategy generally improves generalization performance across a wide range of molecular property prediction tasks.

## Discussion

The results of the WAIC and WBIC analyses show that Graph LDA consistently outperforms the baseline models across a wide range of molecular properties, including quantum chemical, physicochemical, and biochemical properties. Furthermore, analysis of the learned latent representations indicates that Graph LDA yields smoother and more multimodal latent spaces compared with the baseline models.

Our analysis results provide empirical evidence that incorporating a latent diffusion–based prior into molecular representation learning leads to improved generalization performance in molecular property prediction. Beyond reporting generalization performances (WAIC and WBIC scores), our analysis framework reveals that this improvement is closely associated with qualitative changes in the structure of the learned latent space, particularly increased smoothness and multimodality. A latent diffusion–based prior naturally induces these properties in the latent representations of graph VAEs by modeling complex, non-Gaussian latent distributions while maintaining continuity through the diffusion process.

In contrast to existing diffusion models that primarily focus on molecular generation, we explicitly treat diffusion as a learnable prior distribution for molecular representations and analyze its contribution to generalization in downstream prediction tasks. Moreover, unlike latent diffusion VAEs developed in the image domain, Graph LDA is designed specifically for permutation-invariant molecular graph representations, enabling a principled analysis of latent structure rather than focusing solely on improved sample quality.

### Limitations

Although Graph LDA consistently demonstrates superior generalization performance compared with the baseline models on the ZINC-250k dataset, the performance gain is less pronounced on QM9. This difference is likely attributable to the dimensionality of the molecular latent vectors: the latent vectors are 90-dimensional for ZINC-250k, whereas they are 50-dimensional for QM9. These results suggest that latent diffusion priors provide a larger benefit when the molecular latent representation is high-dimensional, where a simple prior (e.g., a standard normal distribution) may impose overly restrictive regularization.

In addition, Graph LDA (stable) is fine-tuned on a pre-trained Graph AE by the multi-stage training, and thus its performance can be influenced by the quality of the pre-trained representation. Addressing this issue may require selecting more appropriate backbone autoencoders for pre-training or developing more effective training schemes beyond the current multi-stage strategy to better decouple the learned representations from the initialization.

Another limitation arises from the backbone autoencoder architecture employed in Graph LDA. Specifically, the PIG-VAE architecture used in this study requires fixing the maximum number of atoms per molecule during both training and inference. As a result, the datasets are restricted to molecules whose sizes do not exceed those in ZINC-250k and QM9. Incorporating a more flexible backbone architecture would enable the model to handle a wider range of molecular sizes and more diverse chemical compounds, thereby improving applicability to larger and more complex molecular systems. Importantly, the analysis perspective adopted in this study is not specific to PIG-VAE and can be readily extended to other backbone autoencoder architectures for molecular representation learning.

## Conclusion

In this study, we systematically investigated the role of latent diffusion in molecular representation learning from the perspective of generalization performance in molecular property prediction. Rather than introducing a new generative architecture, this study focused on establishing an evaluation framework to understand how latent diffusion–based priors influence molecular latent representations and improve generalization performance.

By integrating a latent diffusion-based prior into a permutation-invariant graph VAE, we constructed Graph LDA as a unified framework for unsupervised molecular representation learning and generalization analysis. Through rigorous evaluation using WAIC and WBIC, we demonstrated that molecular representations learned via latent diffusion consistently exhibit superior generalization performance across multiple molecular properties. Furthermore, our quantitative analysis framework for learned molecular representations revealed that this improvement is attributable to enhanced smoothness and multimodality of the learned latent representations, providing mechanistic insight into how diffusion-based priors contribute to generalization.

These findings highlight that diffusion models, when used as latent priors rather than direct generators, play a fundamental role in shaping the geometry of molecular representation spaces. This perspective offers a principled design guideline for future molecular representation learning models aimed at robust generalization, beyond empirical performance gains.

### Future directions

In future works, Graph LDA can be used as a sampler for molecular structures with specific properties by replacing the latent diffusion model with a conditional diffusion model [57, 58]. Integrating the conditional diffusion model with Graph LDA can enable the learning of a suitable latent prior distribution for generating molecular structures with specific properties.

## Supplementary Information

Supplementary material 1.

## Data Availability

The source code and dataset are available from https://github.com/daiki-ko/graph_lda.

## Abbreviations

DNNs: Deep neural networks
GNNs: Graph neural networks
CNN: Convolutional neural network
VAE: Variational autoencoder
ELBO: Evidence lower bound
SMILES: Simplified molecular input line entry system
SELFIES: Self-referencing embedded strings
DDPM: Denoising diffusion probabilistic model
OOD: Out-of-distribution
NLL: Negative log likelihood
FID: Frechet inception distance
FCD: Frechet ChemNet distance
LSGM: Latent score-based generative model
PIG-VAE: Permutation-invariant graph variational autoencoder
MPNN: Message-passing neural networks
AIC: Akaike’s information criterion
BIC: Bayesian information criterion
UMAP: Uniform manifold approximation and projection
KDE: Kernel density estimation
WAIC: Widely applicable information criterion
WBIC: Widely applicable Bayesian information criterion
LMC: Langevin Monte Carlo
ECFP: Extended-connectivity fingerprints
