# Supplementary material for: Graph latent diffusion-based molecular representation learning for enhanced generalization in molecular property prediction
Source: J Cheminform. 2026 Mar 16;18:48. doi: 10.1186/s13321-026-01176-8 (PMC13104421; doi:10.1186/s13321-026-01176-8)
Supplement: Supplementary file 1 — Supplementary material 1. [file 13321_2026_1176_MOESM1_ESM.pdf]

Supplementary Materials for
"Graph Latent Diffusion-based Molecular Representation Learning for Enhanced Generalization in Molecular Property Prediction"

Daiki Koge*[1], Naoaki Ono[2], Takashi Abe[1], Shigehiko Kanaya[3]

1. Department of Electrical and Information Engineering, Graduate School of Science and Technology, Niigata University
2. Faculty of Information and Communication Engineering, Osaka Electro-Communication University
3. Division of Information Science, Graduate School of Science and Technology, Nara Institute of Science and Technology

*Corresponding author e-mail : daiki-ko@ie.niigata-u.ac.jp

1. Permutation-invariant graph variational autoencoder (PIG-VAE)

This section describes the architectures of encoder and decoder in the PIG-VAE.

Encoder: Permutation-invariant graph encoder with self-attention on directional messages.

The PIG-VAE encoder aggregates neighborhood information of atoms via message passing between each atom and its neighboring nodes in a molecular graph based on message-passing neural networks (MPNNs) [1]. For a molecular graph $\mathcal{G} = (\mathcal{V}, \mathcal{E})$, the message vector $\boldsymbol{m}_{ij}$ from the i-th to the j-th atom is defined as

$$\boldsymbol{m}_{ij} = \sigma([\boldsymbol{v}_i \| \boldsymbol{v}_j \| \boldsymbol{e}_{ij}]\mathbf{W}^{(\mathrm{enc})} + \mathbf{b}^{(\mathrm{enc})})$$

where σ is a non-linear activation function, $\|$ represents the concatenation operator, and $\mathbf{W}^{(\mathrm{enc})}$ and $\mathbf{b}^{(\mathrm{enc})}$ are trainable weight parameters. We create a message matrix $\mathbf{W}^{(\mathrm{enc})} \in \mathbb{R}^{n \times n \times d_m}$ with a message vector m_{ij} as the (i, j) element. d_m is the dimension of the message vectors.

In addition, the encoder extracts the relevance between messages using self-attention [2]. An attention head is defined as

$$\mathbf{M}^{(\mathrm{out})} = \mathrm{Attention}(\mathbf{Q}, \mathbf{K}, \mathbf{V}) = \mathrm{softmax}\left(\frac{\mathbf{Q}\mathbf{K}^\top}{\sqrt{d_h}}\right)\mathbf{V}$$

with queries $\mathbf{Q} = \mathbf{M}^{(\mathrm{enc})}\mathbf{W}^Q$; keys $\mathbf{K} = \mathbf{M}^{(\mathrm{enc})}\mathbf{W}^K$; values $\mathbf{V} = \mathbf{M}^{(\mathrm{enc})}\mathbf{W}^V$; and trainable weights $\mathbf{W}^Q \in \mathbb{R}^{d_m \times d_h}$, $\mathbf{W}^K \in \mathbb{R}^{d_m \times d_h}$, and $\mathbf{W}^V \in \mathbb{R}^{d_m \times d_h}$. For multiheaded self-attention, multiple attention heads are concatenated and input to a linear layer with dh output features. Self-attention is repeatedly applied to $\mathbf{M}^{(\mathrm{enc})}$. The message matrix subjected to L iterations of self-attention is denoted as $\mathbf{M}^{(\mathrm{enc},\, L)}$. To encode a molecular graph into a fixed-size, permutation-invariant molecular latent representation, a dummy node $\boldsymbol{v}_0$ is introduced as a gathering node to the input molecular graphs in advance. Message passing and L iterations of self-attention operations are applied to the molecular graph, including the dummy node $\boldsymbol{v}_0$, and information regarding the graph structure is accumulated in $\boldsymbol{m}_{00} \in \mathbf{M}^{(\mathrm{enc},\, L)}$. We sample a molecular latent vector $\boldsymbol{z}_0$ using the reparameterization trick [3] from the following multivariate normal distribution:

$$\boldsymbol{z}_0 \sim \mathcal{N}\left(f_\mu\left(\mathbf{m}_{00}^{(\mathrm{enc},L)}\right), f_\sigma\left(\mathbf{m}_{00}^{(\mathrm{enc},L)}\right)\boldsymbol{I}\right),$$

with $f_\mu: \mathbf{m}_{00} \to \boldsymbol{\mu} \in \mathbb{R}^{d_z}$ and $f_\sigma: \mathbf{m}_{00} \to \boldsymbol{\sigma} \in \mathbb{R}^{d_z}$, parametrized by a linear layer.

Decoder: Permutation-invariant graph decoder with soft-sort operator.

For decoding a molecular graph, PIG-VAE predicts a positional encoding vector $\mathbf{PE}(i)$ that represents the positional information of each atom $\boldsymbol{v}_i$ on the molecular graph. The vector $\mathbf{PE}(i)$ is combined with latent vectors $\boldsymbol{z}_0$. Sinusoidal encoding [4] is used for positional encoding, requiring a permutation π on the input data. However, the permutations of atoms on the molecular graph are ambiguous, and multiple permutations are possible. PIG-VAE sets an arbitrary permutation π for the molecular graph input to the encoder and then rearranges the list of positional encoding vectors $\mathbf{PE}_\pi = [\mathbf{PE}_\pi(1), \ldots \mathbf{PE}_\pi(n)] \in \mathbb{R}^{n \times d_p}$ created with the

permutation π into an appropriate permutation π' to reconstruct the input molecular graph. Although reordering is a non-differentiable operation, PIG-VAE uses a soft-sort operator [5][6] to relax the permutation matrix $\boldsymbol{P}_{\pi\to\pi'}$ for reordering into a continuous matrix $\widehat{\boldsymbol{P}}_{\pi\to\pi'}$. The continuous permutation matrix $\widehat{\boldsymbol{P}}_{\pi\to\pi'}$ is predicted by a soft-sort operator based on $\mathbf{m}_{ii}^{(\mathrm{enc},L)} \in \mathrm{diag}\left(\mathbf{M}^{(\mathrm{enc},L)}\right)$ for $i > 0$.

In decoding a molecular graph, the initial message vector $\mathbf{m}_{ij}^{(\mathrm{dec},0)} \in \mathbf{M}^{(\mathrm{dec},0)}$ is computed as follows, using a linear transformation $\left(\widehat{\mathbf{P}}_{\pi\to\pi'}\mathbf{PE}_{\pi}\right)$ with $\widehat{\boldsymbol{P}}_{\pi\to\pi'}$.

$$\mathbf{m}_{ij}^{(\mathrm{dec},0)} = \sigma\left(\left[\boldsymbol{z}_0 + [\mathbf{PE}_{\pi'}(i) \| \mathbf{PE}_{\pi'}(j)]\right]\mathbf{W}^{(\mathrm{dec})} + \mathbf{b}^{(\mathrm{dec})}\right),$$

where $\mathbf{W}^{(\mathrm{dec})}$ and $\mathbf{b}^{(\mathrm{dec})}$ are learnable parameters. After L iterations of self-attention on the message matrix $\mathbf{W}^{(\mathrm{dec},0)}$, we extract atom features $\boldsymbol{v}_i \in \mathcal{V}$ and bond features $\boldsymbol{e}_{ij} \in \mathcal{E}$ by a final layer from each message vector $\mathbf{m}_{ij}^{(\mathrm{dec},0)} \in \mathbf{W}^{(\mathrm{dec},L)}$:

$$\boldsymbol{v}_i = \mathrm{softmax}\left(\mathbf{m}_{ii}^{(\mathrm{dec},L)}\mathbf{W}_v + \mathbf{b}_v\right)$$

$$\boldsymbol{e}_{ij} = \mathrm{softmax}\left(0.5 \cdot \left(\mathbf{m}_{ij}^{(\mathrm{dec},L)} + \mathbf{m}_{ji}^{(\mathrm{dec},L)}\right)\mathbf{W}_e + \mathbf{b}_e\right),$$

where $\mathbf{W}_v \in \mathbb{R}^{d_h \times d_v}$, $\mathbf{W}_e \in \mathbb{R}^{d_h \times d_e}$, $\mathbf{b}_v \in \mathbb{R}^{d_v}$, and $\mathbf{b}_e \in \mathbb{R}^{d_e}$ denote the trainable parameters.

2. Latent diffusion model

Table S1 summarizes the final selection of hyperparameters for the training of the latent diffusion model.

Table S1: Hyperparameters for the latent diffusion model.

Description	Models for ZINC-250k		Models for QM9	
	Graph LDA	Graph LDA (stable)	Graph LDA	Graph LDA (stable)
1. Time encoding function	Sinusoidal encoding [4]	Sinusoidal encoding	Sinusoidal encoding	Sinusoidal encoding
2. Dimension of time embeddings	256	256	256	256
3. Number of time steps for diffusion	200	1,000	100	500
4. Dimension of hidden feature for noisy latent vectors $\boldsymbol{z}_t$	256	256	256	256
5. Number of layers	3	3	3	3
6. Learning rate	0.0005	2nd stage in Algorithm 1: 0.0001, 3rd stage in Algorithm 1: 0.0001.	0.0005	2nd stage in Algorithm 1 : 0.0005, 3rd stage in Algorithm 1 : 0.0001.

3. Bayesian neural network for WAIC and WBIC evaluation (Section 3.3.1)

Bayesian neural networks are established to calculate the WAIC and WBIC values of the molecular property predictive models. Tables S2, S3 and S4 summarizes the final selection of hyperparameters for these networks. Hyperparameters (a) in Table S3 are used for predicting BACE, CTSD, MMP2, Malaria, ESOL, Lipo, LogP, and Freesolv, while the hyperparameters (b) in Table S4 are used for predicting HOMO and LUMO.

Table S2: Hyperparameters of Bayesian neural networks for unsupervised learning models.

Description	Pre-trained models by ZINC-250k	Pre-trained models by QM9
Dimension of input feature	90	50
Dimension of hidden feature	256	128
Number of layers	3	3
Activation function	Tanh	Swish [7]

Table S3: Hyperparameters (a) of Bayesian neural networks for SSL models.

Description	MolCLR	Graph MVP	3D-Infomax
Dimension of input feature	256	300	256
Dimension of hidden feature	128	256	128
Number of layers	3	3	3
Activation function	Tanh	Tanh	Tanh

Table S4: Hyperparameters (b) of Bayesian neural networks for SSL models.

Description	MolCLR	Graph MVP	3D-Infomax
Dimension of input feature	256	300	256
Dimension of hidden feature	128	256	128
Number of layers	3	3	3
Activation function	Swish	Swish	Swish

4. Hyperparameters of LMC for calculating WAIC and WBIC

WAIC and WBIC values are calculated by LMC. Tables S5, S6 and S7 summarize the final selection of hyperparameters for LMC. The hyperparameters (a) in Table S6 are used for the prediction models of BACE, CTSD, MMP2, Malaria, ESOL, Lipo, LogP, and Freesolv, while the hyperparameters (b) in Table S7 are used for the prediction models of HOMO and LUMO.

Table S5: Hyperparameters of LMC for unsupervised learning models.

Description	Pre-trained models by ZINC-250k		Pre-trained models by QM9	
	WAIC	WBIC	WAIC	WBIC
Step size (η)	0.001	0.01	0.001	0.01
Number of iterations	5000	3000	5000	3000
Burn-in period	2000	1000	2000	1000

Table S6: Hyperparameters (a) of LMC for SSL models.

Description	MolCLR		Graph MVP		3D-Infomax	
	WAIC	WBIC	WAIC	WBIC	WAIC	WBIC
Step size (η)	0.001	0.01	0.001	0.01	0.001	0.01
Number of iterations	5000	3000	5000	3000	5000	3000
Burn-in period	2000	1000	2000	1000	2000	1000

Table S7: Hyperparameters (b) of LMC for SSL models.

Description	MolCLR		Graph MVP		3D-Infomax	
	WAIC	WBIC	WAIC	WBIC	WAIC	WBIC
Step size (η)	0.001	0.001	0.001	0.001	0.001	0.001
Number of iterations	5000	5000	5000	5000	5000	5000
Burn-in period	2000	2000	2000	2000	2000	2000

5. Generalization performance of unsupervised learning models for HOMO and LUMO properties without 3D geometry.

Table S8: Generalization performance of unsupervised learning models.

Models	HOMO		LUMO	
	WAIC	WBIC	WAIC	WBIC
Graph AE	0.9198	4606.07	0.9205	4604.31
Graph VAE	0.9202	4620.27	0.9203	4613.73
Graph Flow	0.9193	4609.03	0.9201	4607.47
Graph LDA	0.9193	4621.76	0.9201	4601.51
Graph LDA (stable)	0.9193	4601.12	0.9202	4602.81

6. Predictive Performance of Fine-tuned Models

For evaluating the predictive performance of each model, we employ a hold-out evaluation with multiple random splits. Specifically, the data are randomly divided into 80/10/10 train/validation/test splits, and the pre-trained encoder is fine-tuned for 200 epochs on each molecular property dataset. This procedure is repeated over four independent random splits, and the test performance is averaged over four independent random splits. Table S9 summarizes the predictive performance of Graph LDA and baseline models. BACE, CTSD, MMP2, Malaria, ESOL, Freesolv, Lipo, and LogP are evaluated using root-mean-square error (RMSE), while HOMO and LUMO are evaluated using mean absolute error (MAE), following the MoleculeNet benchmark protocol. Graph LDA (stable) achieves the lowest error in 5 out of the 10 properties and maintains highly competitive performance for the others. Furthermore, when restricting the comparison to autoencoder-based unsupervised learning models (i.e., Graph AE, Graph VAE, and Graph Flow), Graph LDA (stable) generally shows competitive or lower error values across many tasks.

Table S9: Predictive performance of fine-tuned models (test set).

Molecular Property	Models							
	Graph AE	Graph VAE	Graph Flow	Graph LDA	Graph LDA (Stable)	MolCLR	Graph MVP	3D-infomax
BACE (502)	0.71641	0.75766	0.73785	0.74362	<u>**0.71566**</u>	1.23358	0.78697	0.89661
CTSD (84)	0.46309	0.48346	0.52779	<u>**0.43952**</u>	0.44769	0.84050	0.53319	0.85343
MMP2 (1046)	0.81910	0.77775	<u>**0.75860**</u>	0.80459	0.80457	1.35236	0.88345	0.90198
Malaria (3019)	1.30500	1.30280	1.32346	1.32580	<u>1.28161</u>	1.42076	**1.26835**	1.33409
ESOL (1080)	0.60842	0.60610	0.62596	0.62770	<u>**0.59440**</u>	1.47215	0.76750	0.75599
Freesolv (642)	0.93793	1.13261	1.00666	0.90756	<u>**0.88861**</u>	2.30463	1.47794	1.48882
Lipo (1903)	0.71783	0.74269	0.74541	0.71280	<u>**0.69853**</u>	1.10715	0.70189	0.72049
LogP (100k)	0.07103	0.07201	<u>**0.06257**</u>	0.07129	0.07480	0.17689	0.11502	0.18518
HOMO (100k)	0.00611	0.00580	0.00607	0.00619	<u>**0.00568**</u>	0.01323	0.00643	0.00689
LUMO (100k)	<u>**0.00566**</u>	0.00571	0.00617	0.00601	0.00605	0.02327	0.00692	0.00636

Note : The best predictive performance (i.e., the lowest error) across all models is highlighted in bold, while the best-performing autoencoder-based unsupervised learning model is underlined.

7. kNN rate and hyperparameters of UMAP

Figures S1 and S2 show the kNN-rate curves for different dimensions of the low-dimensional space of UMAP. The kneedle algorithm [8] detects the elbow point from the distance between the line ($y = ax + b$) connecting the start and end points of the curve and kNN-rate of each point. The green line in both figures represents the distance, and the elbow point corresponds to the maximum distance.

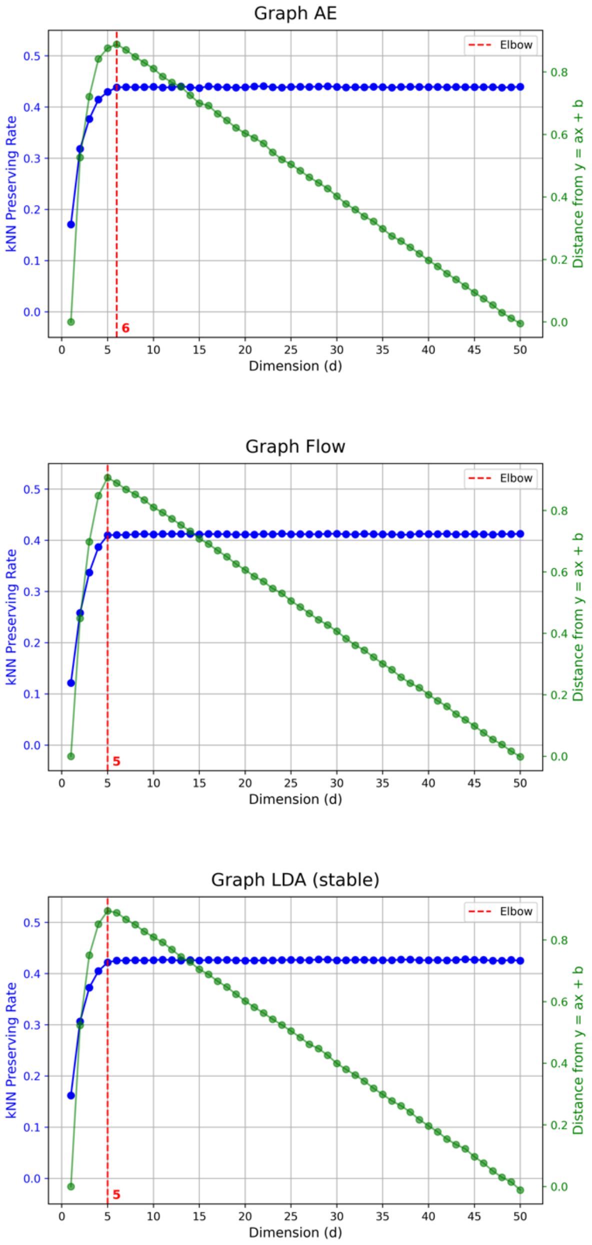

Figure S1: kNN-rate for each model on the ZINC-250k dataset.

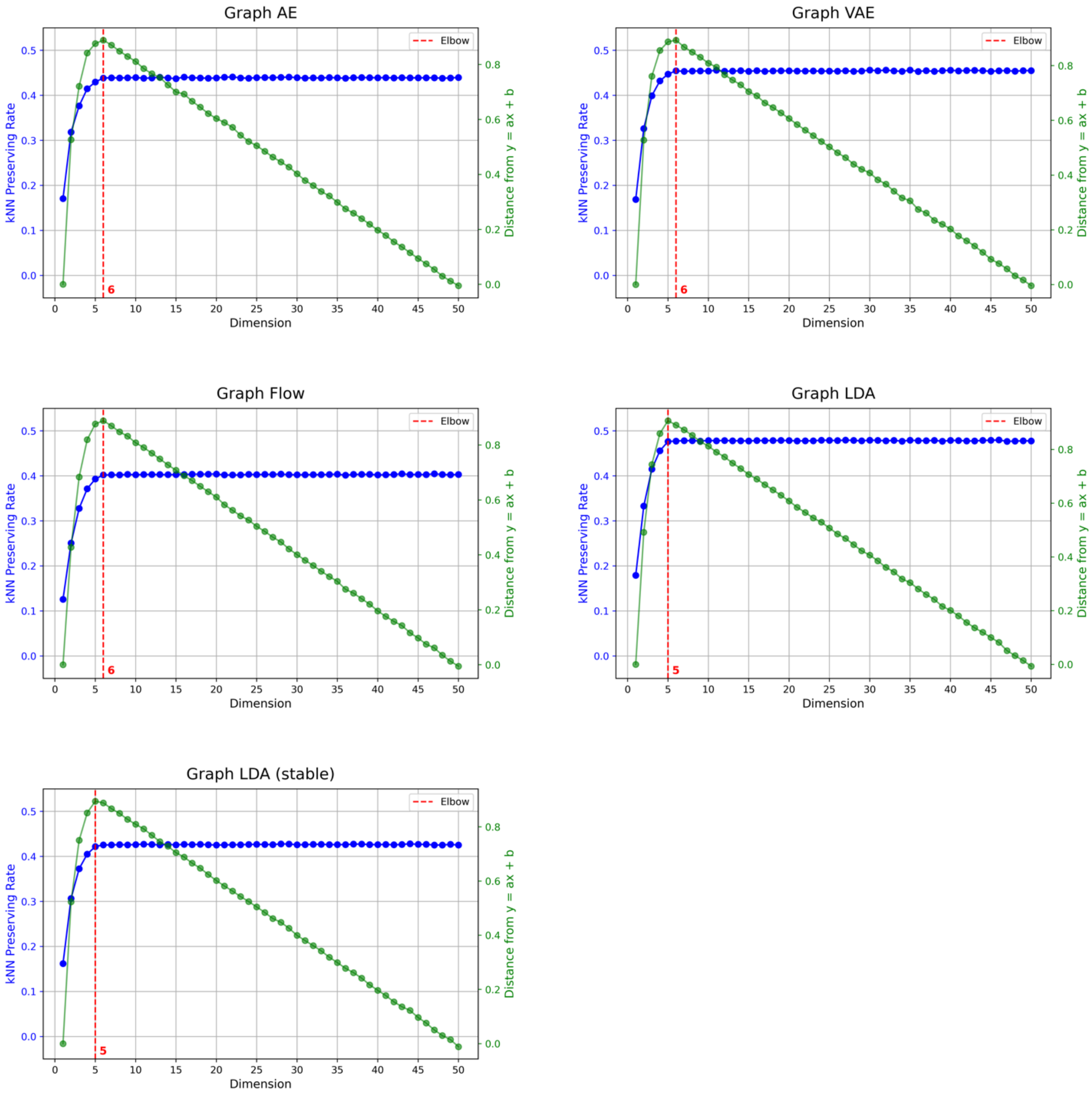

Figure S2: kNN-rate for each model on the QM9 dataset.

8. Smoothness of molecular latent representations

Table S10 lists the smoothness values pertaining to the molecular latent representations for each model.

Table S10: Comparison of smoothness between the proposed and baseline models.

Molecular Property	Models				
	Graph AE	Graph VAE	Graph Flow	Graph LDA	Graph LDA (Stable)
BACE (502)	0.8266	1.6432	1.0107	0.9329	<u>0.8000</u>
CTSD (84)	<u>0.4689</u>	0.7315	0.5970	0.5196	0.4865
MMP2 (1046)	1.0401	2.0042	1.2032	1.1440	<u>1.0203</u>
Malaria (3019)	1.2847	1.6487	1.3436	1.3372	<u>1.2842</u>
ESOL (1080)	1.1205	2.0684	1.2020	1.3619	<u>1.1161</u>
Freesolv (642)	2.1040	3.8106	<u>2.0970</u>	2.3131	2.1900
Lipo (1903)	1.0372	1.4284	1.1504	1.1743	<u>1.0313</u>
LogP (100k)	<u>0.9086</u>	1.6703	1.0990	1.0432	0.9312
HOMO (100k)	<u>1.5727×10^{-2}</u>	1.5863×10^{-2}	1.6253×10^{-2}	1.6628×10^{-2}	1.5960×10^{-2}
LUMO (100k)	3.2387×10^{-2}	3.1409×10^{-2}	3.1813×10^{-2}	3.5626×10^{-2}	<u>3.0913×10^{-2}</u>

9. Computational details

OS: Red Hat Enterprise Linux release 8.4
CPU: Intel(R) Xeon(R) Gold 6230R CPU @ 2.10GHz
GPU: NVIDIA Corporation GA100 [A100 PCIe 40GB]
All experiments are conducted using Python 3.10.13. RDKit (version 2024.3.5.0) is used to treat the organic compound data, and PyTorch (version 2.4.0) and Pytorch Geometric (version 2.5.3) are used as the deep learning frameworks.

Tables S11 and S12 summarize the computational times for training Graph VAE and Graph LDA.

Table S11: Computational time for ZINC-250k dataset with GPU.

Description	Graph VAE	Graph LDA (stable)		
		1. Pre-training of Graph AE	2. Training of Latent DDPM	3. Training of Graph LDA
Training time per epoch (min)	19.5	19.5	0.1	19.8
Number of epochs	100	100	500	100
Total training time (min)	1950	3980		

Table S12: Computational time for QM9 dataset with GPU.

Description	Graph VAE	Graph LDA (stable)		
		1. Pre-training of Graph AE	2. Training of Latent DDPM	3. Training of Graph LDA
Training time per epoch (min)	19.45	19.45	0.1	19.75
Number of epochs	100	100	500	100
Total training time (min)	1945	3970		

10. Comparison of generalization performance between joint and multi-stage training models

Table S13 summarizes the generalization performance of the Graph LDA models trained by the conventional joint-training scheme and the proposed multi-stage training scheme.

Table S13: Generalization comparison of the Graph LDA models trained by the joint and multi-stage training schemes.

Molecular property	Joint Training Model		Multi-Stage Training Model	
	WAIC	WBIC	WAIC	WBIC
BACE (502)	1.6649	851.86	1.5737	843.67
CTSD (84)	1.5114	122.64	1.5975	124.98
MMP2 (1046)	2.2960	2426.64	1.8984	2079.20
Malaria (3019)	1.9622	6081.41	1.8790	6086.26
ESOL (1080)	2.4819	2686.33	1.9891	2223.51
Freesolv (642)	6.2410	3829.41	7.4011	2845.08
Lipo (1903)	1.6742	3213.24	1.5657	3068.35
LogP (5000)	1.5379	8189.20	1.2606	6532.18
HOMO (5000)	0.9194	4601.64	0.9193	4602.16
LUMO (5000)	0.9201	4604.51	0.9201	4604.07

References

[1] Gilmer, J., Schoenholz, S.S., Riley, P.F., Vinyals, O., Dahl, G.E.: Neural message passing for quantum chemistry. In: International Conference on Machine Learning, pp. 1263–1272 (2017). PMLR

[2] Vaswani, A., Shazeer, N., Parmar, N., Uszkoreit, J., Jones, L., Gomez, A.N., Kaiser, L., Polosukhin, I.: Attention is all you need. Advances in neural information processing systems 30 (2017)

[3] Kingma, D.P., Welling, M.: Auto-encoding variational bayes. arXiv preprint arXiv:1312.6114 (2013)

[4] Vaswani, A., Shazeer, N., Parmar, N., Uszkoreit, J., Jones, L., Gomez, A.N., Kaiser, L., Polosukhin, I.: Attention is all you need. Advances in neural information processing systems 30 (2017)

[5] Prillo, S., Eisenschlos, J.: Softsort: A continuous relaxation for the argsort operator. In: International Conference on Machine Learning, pp. 7793–7802 (2020). PMLR

[6] Grover, A., Wang, E., Zweig, A., Ermon, S.: Stochastic optimization of sorting networks via continuous relaxations. arXiv preprint arXiv:1903.08850 (2019)

[7] Ramachandran P, Zoph B, Le QV (2017) ‘Searching for activation functions.’ *arXiv preprint arXiv:1710.05941*

[8] Satopaa V, Albrecht J, Irwin D, Raghavan B (2011) ‘Finding a’ kneedle in a haystack: Detecting knee points in system behavior." In: 31st International Conference on Distributed Computing Systems Workshops. IEEE, p166–171
